# Supplementary material for: Multi-platform biomarkers of response to an immune checkpoint inhibitor in the neoadjuvant I-SPY 2 trial for early-stage breast cancer
Source: Cell Rep Med. 2024 Nov 6;5(11):101799. doi: 10.1016/j.xcrm.2024.101799 (PMC11604542; doi:10.1016/j.xcrm.2024.101799)
Supplement: Document S1. Figures S1–S7 and Tables S1, S3, and S4 [file mmc1.pdf]

Cell Reports Medicine, Volume *5*

Supplemental information

Multi-platform biomarkers of response to an immune checkpoint inhibitor in the neoadjuvant I-SPY 2 trial for early-stage breast cancer

Michael J. Campbell, Denise M. Wolf, Christina Yau, Lamorna Brown-Swigart, Julie Wulfkuhle, Isela R. Gallagher, Zelos Zhu, Jennifer Bolen, Scott Vandenberg, Clifford Hoyt, Hidetoshi Mori, Alexander Borowsky, Laura Sit, Jane Perlmutter, Smita M. Asare, I-SPY2 Investigators, Rita Nanda, Minetta C. Liu, Douglas Yee, Angela M. DeMichele, Nola M. Hylton, Lajos Pusztai, Donald A. Berry, Gillian L. Hirst, Emanuel F. Petricoin, Laura van't Veer, and Laura Esserman

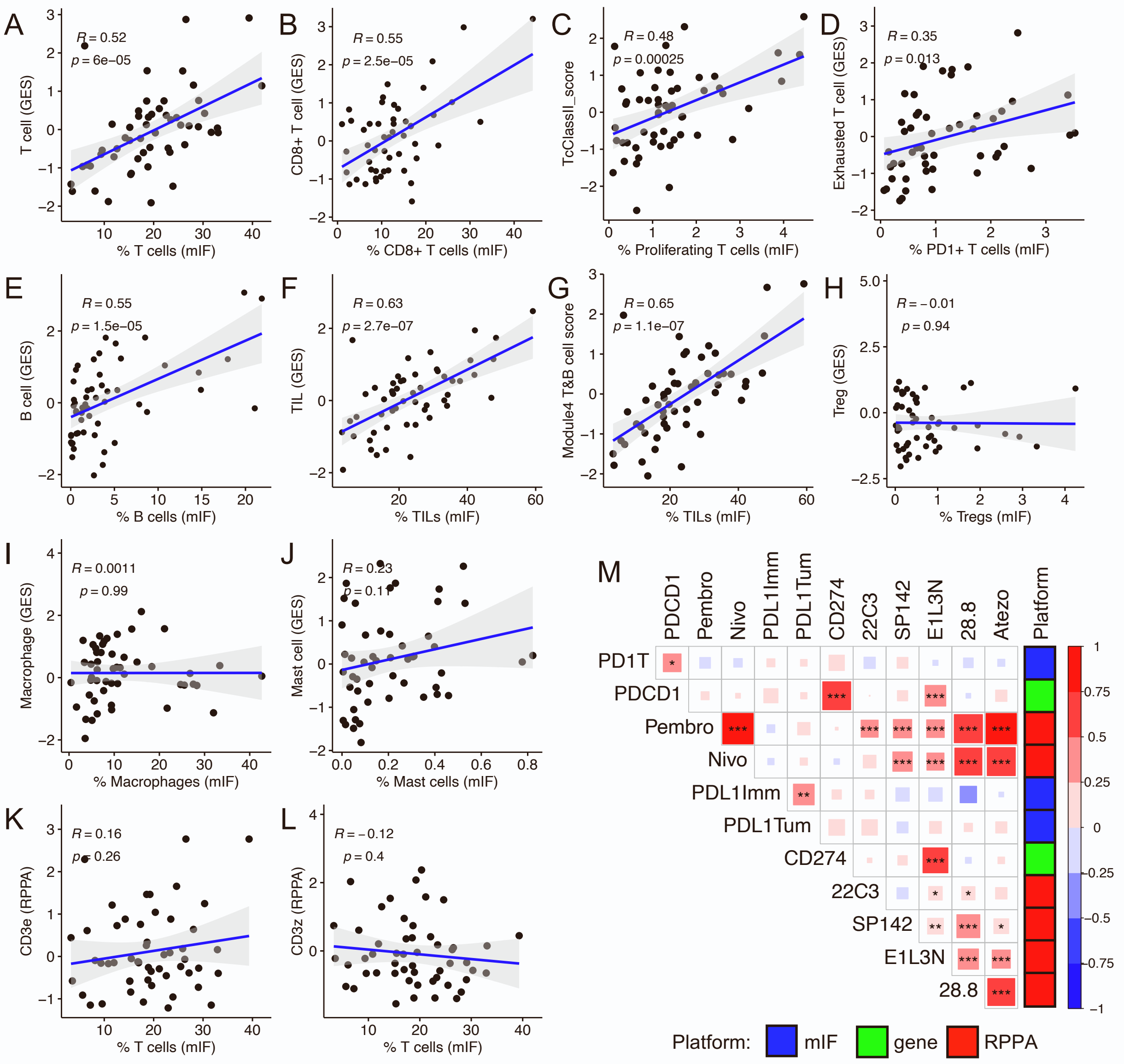

Figure S1. Correlations of immune cell densities measured on different platforms. Related to Figure 1. (A-J) Correlations of immune cells identified by mIF vs gene expression signatures (GES). (K-L) Correlations of immune cells identified by mIF vs RPPA. (M) Correlation matrix of PD-1/PD-L1 biomarkers. Pearson correlations; *p<0.01, **p<0.001, ***p<0.0001. Red square indicate positive correlation, blue squares indicate negative correlation. mIF markers --- PD1T: PD-1^{+} T cells; PDL1Imm: PD-L1^{+} immune cells; PDL1Tum: PD-L1^{+} tumor cells. Gene expression markers --- PDCD1: PD-1 gene; CD274: PD-L1 gene. RPPA markers --- Pembro (pembrolizumab) and Nivo (nivolumab): anti-PD-1 mAbs; 22C3, SP142, E1L3N, 28.8 and Atezo (atezolizumab): anti-PD-L1 mAbs.

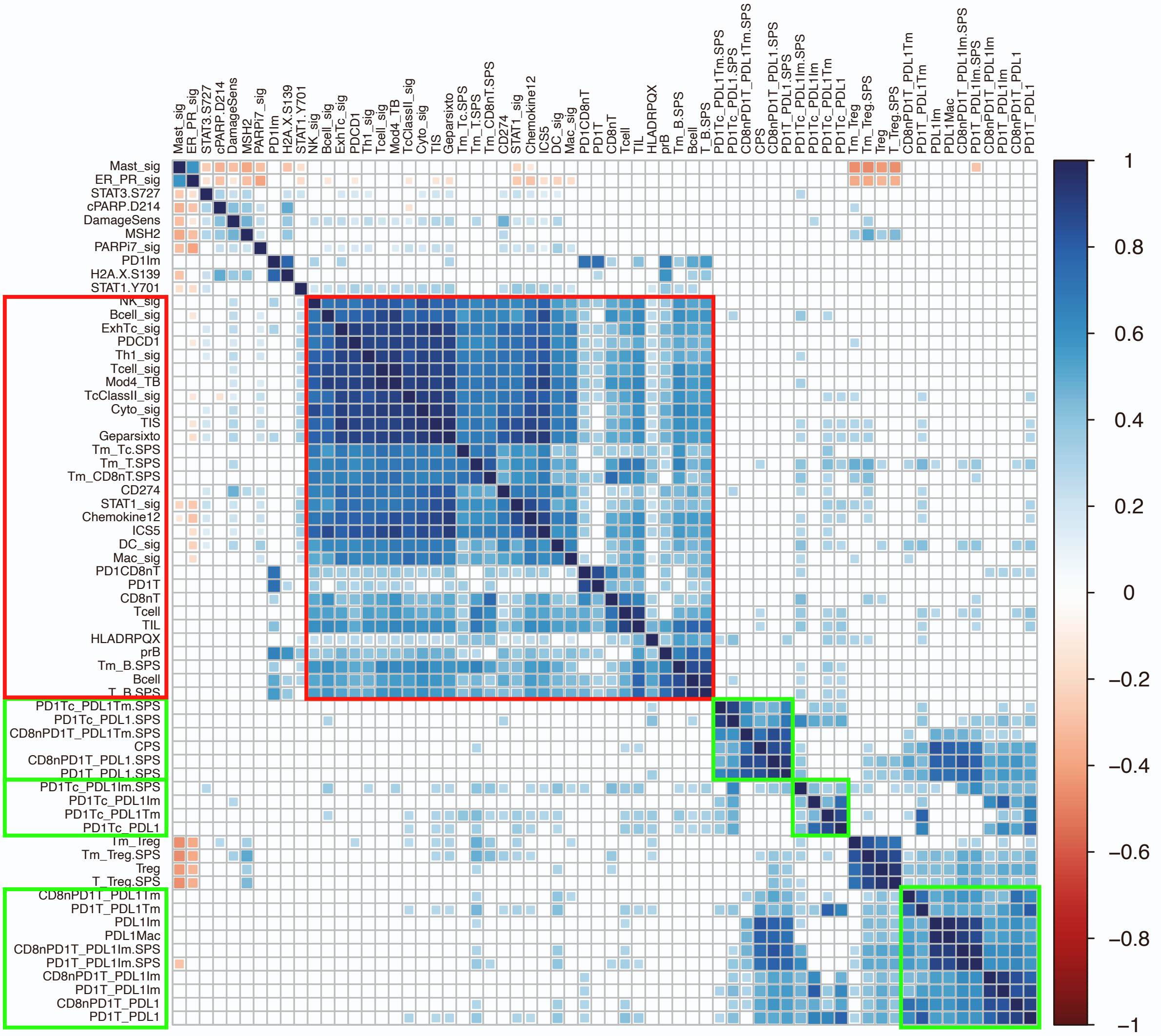

Figure S2. **Correlation matrix of biomarkers significantly associated with pCR in the pembro arm. Related to Figures 3 and 6 and Tables S2-S6.** Significant correlations (Pearson correlations, $p<0.05$) are shown in blue (positive correlation) or red (negative correlation). Red boxed area indicates cluster of immune cell gene expression signatures, immune cell densities by mIF, and immune cell population expression signatures. Green boxes indicate clusters of PD-1/PD-L1 related spatial metrics.

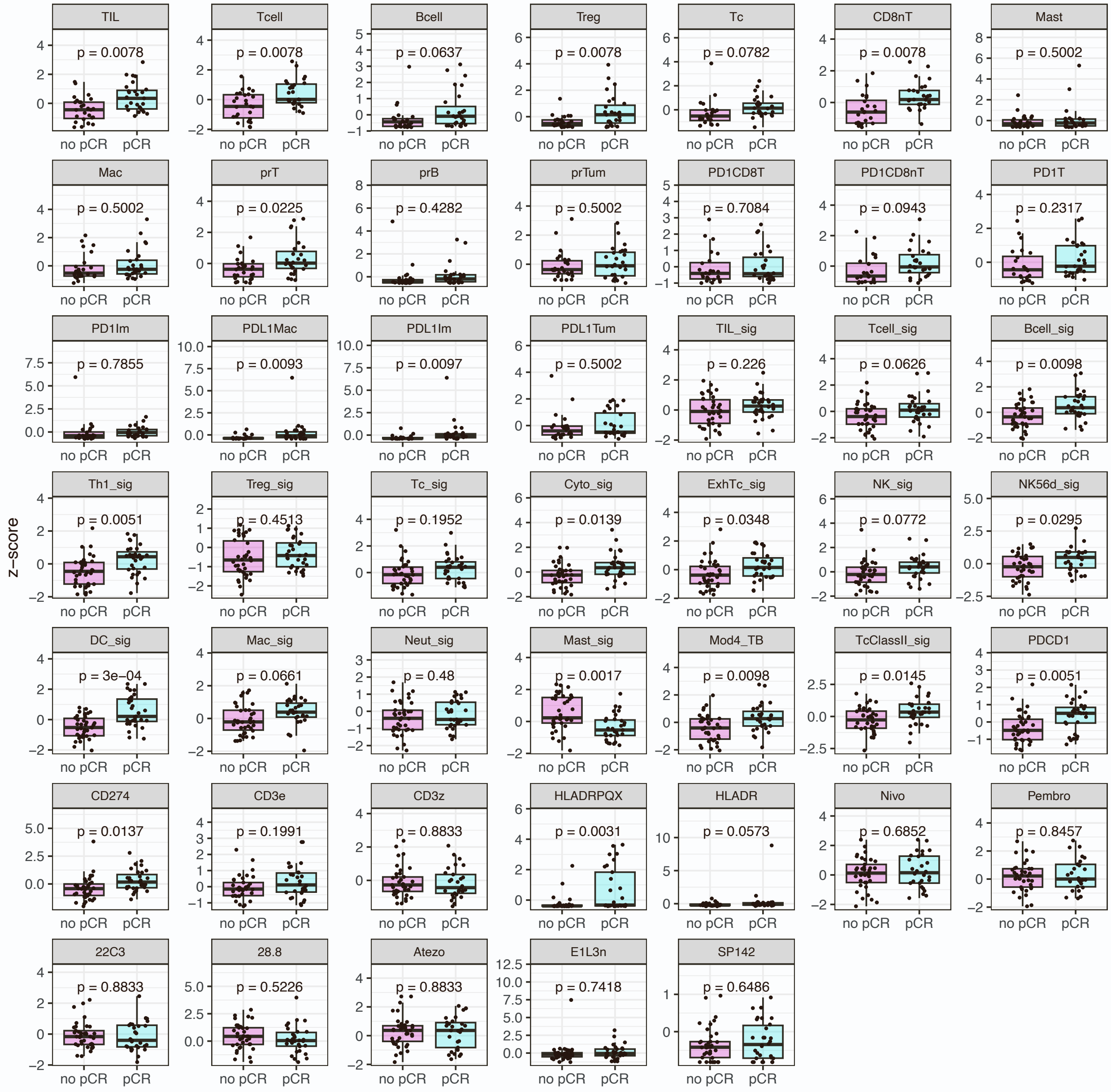

Figure S3. Associations of immune cell markers/populations identified by mIF, gene expression, or RPPA with response to ICB. Related to Figure 3 and Tables S2-S6. Boxplots illustrating the associations of immune cell densities (z-scored cell percentages), immune genes and gene signatures, and RPPA immune markers with response. Descriptions of mIF populations, gene signatures, and RPPA markers are given in Tables S2-S4. The data are depicted as individual dots for each sample, along with the median, first and third quartile. Statistical analysis was performed using the likelihood-ratio test. BH corrected p values shown.

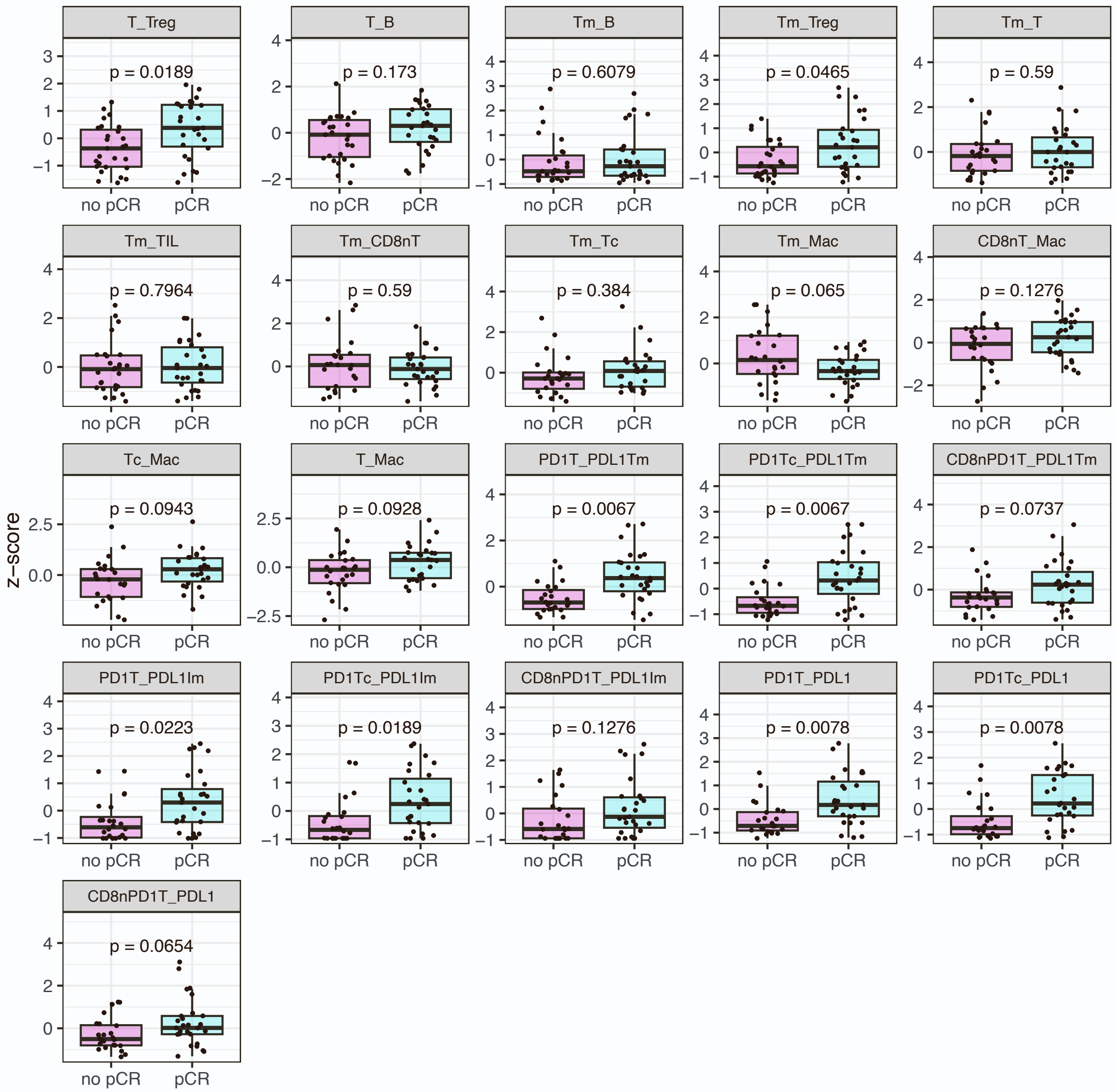

Figure S4. Associations of spatial colocalization of various cell types in the tumor microenvironment with response to ICB. Related to Figure 4 and Tables S4 and S5. Boxplots illustrating the associations of colocalization of cell pairs (Morisita-Horn indices; z-scored) with response. Descriptions of cell pairs are given in Table S4. The data are depicted as individual dots for each sample, along with the median, first and third quartile. Statistical analysis was performed using the likelihood-ratio test. BH corrected p values shown.

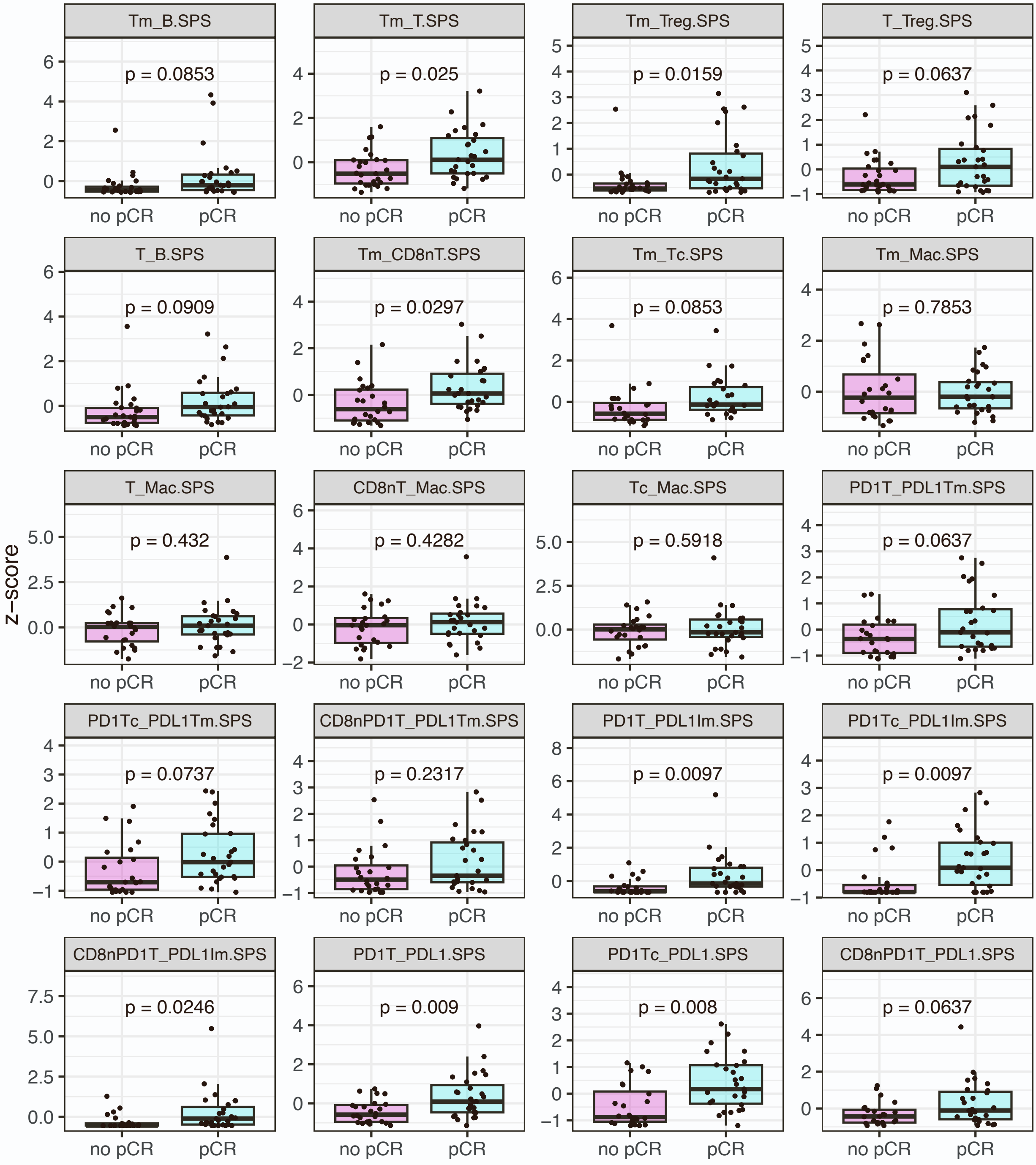

Figure S5. Associations of spatial proximity of various cell types in the tumor microenvironment with response to ICB. Related to Figure 5 and Tables S4 and S5. Boxplots illustrating the associations of spatial proximity scores (SPS) between cell pairs with response. Descriptions of cell pairs analyzed are given in Table S4. The data are depicted as individual dots for each sample, along with the median, first and third quartile. Statistical analysis was performed using the likelihood-ratio test. BH corrected p values shown.

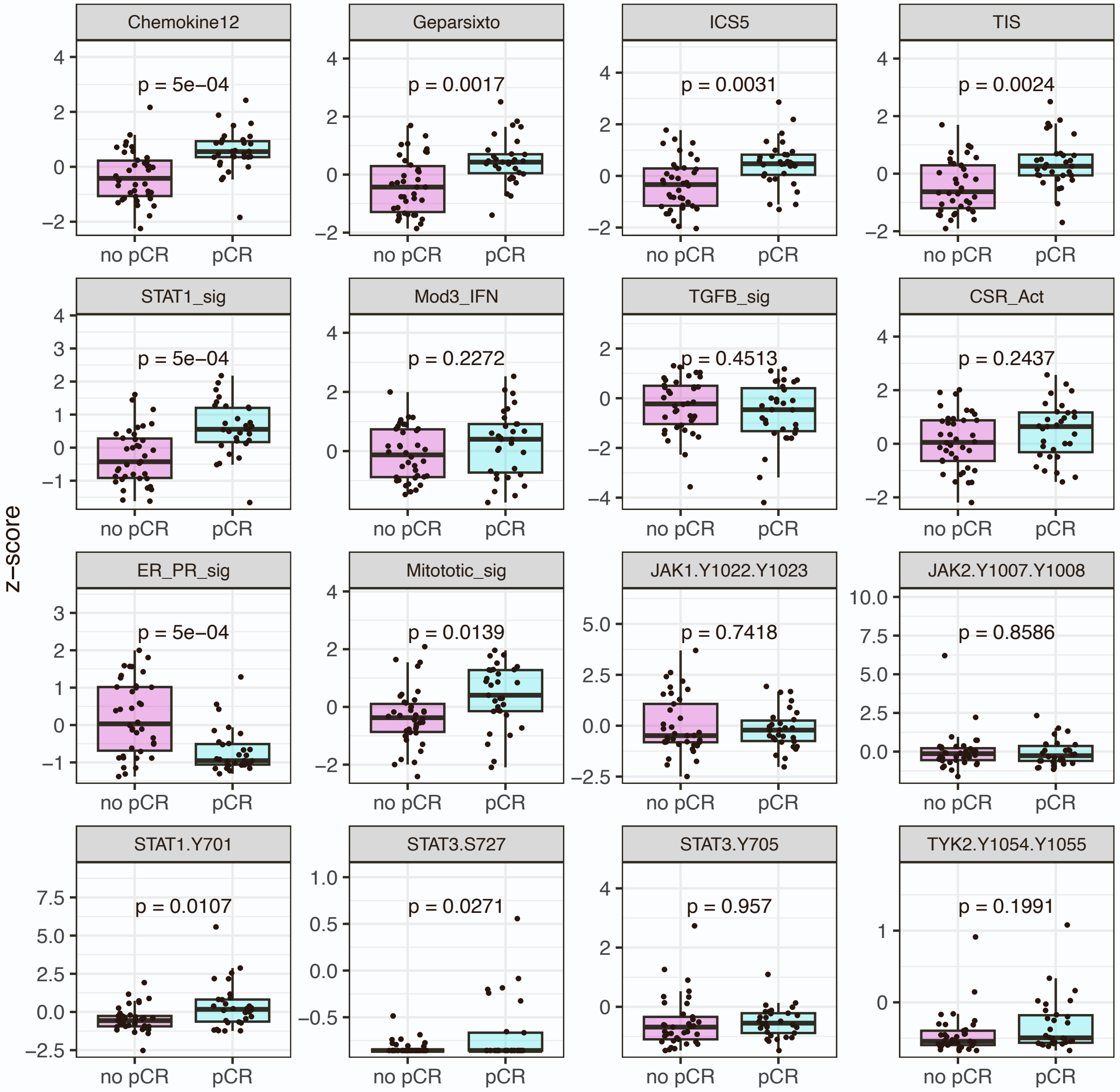

Figure S6. Associations of immune signaling pathways with response to ICB. Related to Figure 6 and Tables S2, S3, and S6. Boxplots illustrating the associations of signaling pathways measured by gene expression or RPPA with response. Descriptions of gene signatures are given in Table S2. Descriptions of RPPA markers are given in Table S3. The data are depicted as individual dots for each sample, along with the median, first and third quartile. Statistical analysis was performed using the likelihood-ratio test. BH corrected p values shown.

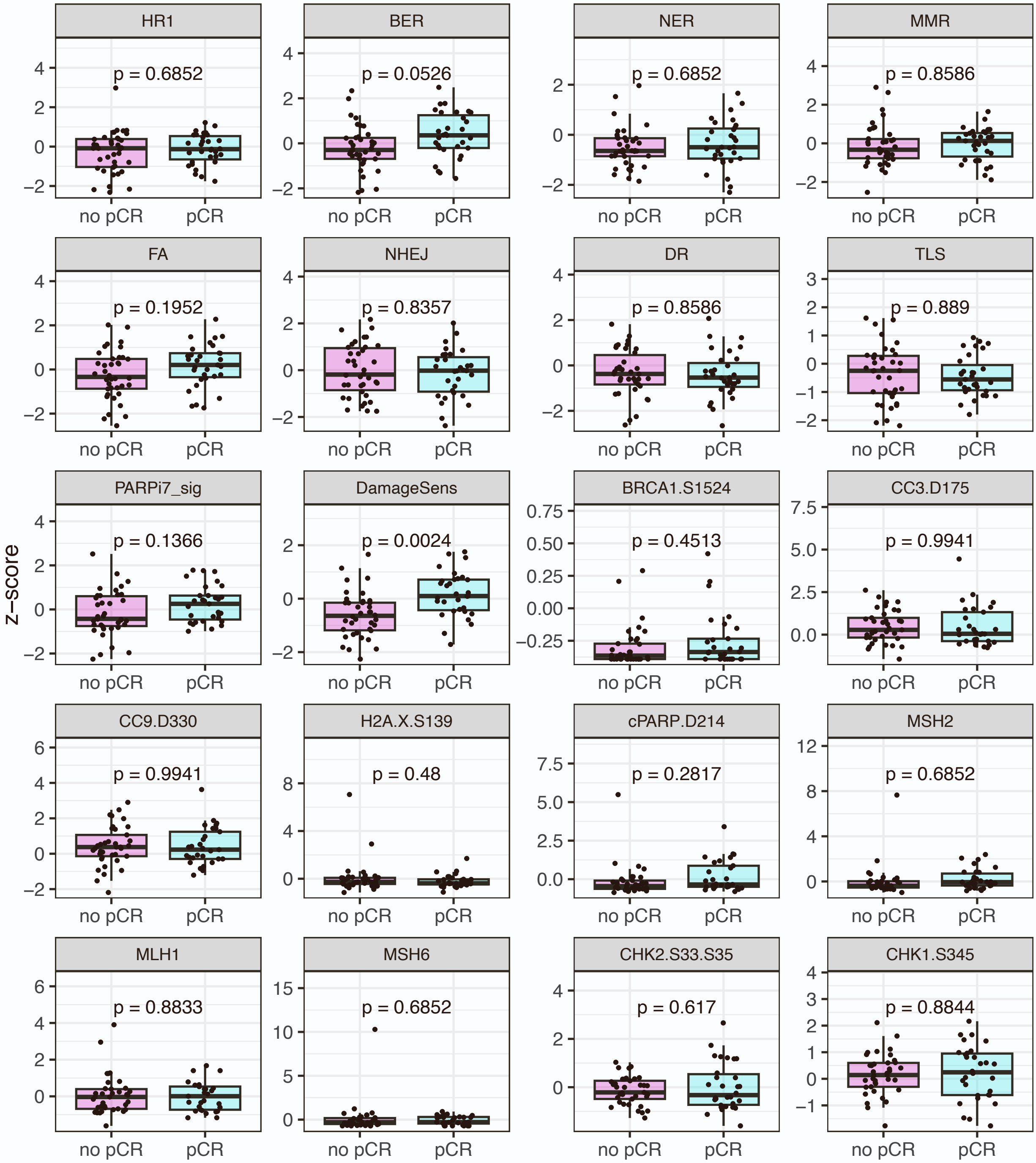

Figure S7. Associations of DNA damage and repair biomarkers with response to ICB. Related to Figure 6 and Tables S2, S3, and S6. Boxplots illustrating the associations of DNA damage and repair biomarkers measured by gene expression or RPPA with response. Descriptions of gene signatures are given in Table S2. Descriptions of RPPA markers are given in Table S3. The data are depicted as individual dots for each sample, along with the median, first and third quartile. Statistical analysis was performed using the likelihood-ratio test. BH corrected p values shown.

Supplemental Table S1. Details of primary antibodies and Opal fluorophores used in mIF. Related to Figure 2 and STAR Methods.

Panel 1

Marker	Clone	Antibody source	Opal TSA	RRID
FoxP3; regulatory T cell marker	SP97	Spring Biosciences	620	AB_10658507
pan-cytokeratin; epithelial cell marker	AE1/AE3	Dako	650	AB_2631307
CD20; B lymphocyte marker	L26	Ventana/Roche	540	AB_2335956
CD3; T lymphocyte marker	2GV6	Ventana/Roche	520	AB_2335978
Ki67; proliferation marker	30-9	Ventana/Roche	570	AB_2631262
CD117; c-Kit, mast cells	D3W6Y	Cell Signaling	690	AB_2799120

Panel 2

Marker	Clone	Antibody source	Opal TSA	RRID
PD-L1; immune checkpoint	E1L3N	Cell Signaling	620	AB_2687655
PD-1; immune checkpoint	EPR4877	Abcam	650	AB_2894867
CD8; cytotoxic T cell marker	4B11	Leica	690	AB_442068
pan-cytokeratin; epithelial cell marker	AE1/AE3	Dako	570	AB_2631307
CD68; macrophage marker	PG-M1	Dako	540	AB_2074844
CD3; T cell marker	2GV6	Ventana/Roche	520	AB_2335978

Supplemental Table S3. Reverse Phase Protein Array (RPPA) based biomarkers evaluated in this study. Related to Figures 3, 6, S2, S3, S6, S7, Table S6, and STAR Methods. DDR: DNA Damage Response.

Biomarker	Type	Description
CD3ε	cell population	CD3 epsilon chain
CD3ζ	cell population	CD3 zeta chain
HLADR	cell population	MHC class II moleculel; HLA-DR
HLADRPQX	cell population	MHC class II molecules; HLA-DR, HLA-DP, HLA-DQ, HLA-DX
28.8	cell population	PD-L1 (antibody clone 28.8)
Atezo	cell population	PD-L1 (atezolizumab)
SP142	cell population	PD-L1 (antibody clone SP142)
E1L3N	cell population	PD-L1 (antibody clone E1L3N)
22C3	cell population	PD-L1 (antibody clone 22C3)
Pembro	cell population	PD-1 (pembrolizumab)
Nivo	cell population	PD-1 (nivolumab)
STAT1.Y701	Immune signaling	Phospho-STAT1; signal transducer and activator of transcription 1
STAT3.S727	Immune signaling	Phospho-STAT3; signal transducer and activator of transcription 3
STAT3.Y705	Immune signaling	Phospho-STAT3; signal transducer and activator of transcription 3
JAK1.Y1022.Y1023	Immune signaling	Phospho-JAK1; Janus kinase 1
JAK2.Y1007.Y1008	Immune signaling	Phospho-JAK2; Janus kinase 2
TYK2.Y1054.Y1055	Immune signaling	Phospho-TYK2; JAK family non-receptor tyrosine protein-kinase
BRCA1.S1524	DDR	Phospho-BRCA1; breast cancer type 1 susceptibility protein
CC3.D175	DDR	Phospho-CC3; cleaved caspase 3
CC9.D330	DDR	Phospho-CC9; cleaved caspase 9
H2A.X.S139	DDR	Phospho-H2A.X; H2A histone family member X
cPARP.D214	DDR	Phospho-cleaved PARP; poly (ADP-ribose) polymerase
MSH2	DDR	MutS homolog 2, DNA mismatch repair protein
MSH6	DDR	MutS homolog 6, DNA mismatch repair protein
MLH1	DDR	MutL homolog 1, DNA mismatch repair protein
CHK1.S345	DDR	Phospho-CHK1, checkpoint kinase 1
CHK2.S33.S35	DDR	Phospho-CHK2, checkpoint kinase 2

Table S4. mIF based biomarkers evaluated in this study. Related to Figures 3, 4, 5, S2, S3, S4, S5, Table S5, and STAR Methods.

Biomarker	Type	Description
Tcell	cell population	T cells (CD3+)
Treg	cell population	Regulatory T cells (CD3+Foxp3+)
Bcell	cell population	B cells (CD20+)
Mast	cell population	Mast cells (CD117+ CK-)
TIL	cell population	Tumor infiltrating lymphocytes (Tell + Bcell)
Tc	cell population	Cytotoxic T cells (CD8+CD3+)
CD8nT	cell population	CD8-negative T cells (CD8-CD3+)
Mac	cell population	Macrophages (CD68+CK-)
prT	cell population	proliferating T cells (Ki67+CD3+)
prB	cell population	proliferating B cells (Ki67+CD20+)
prTum	cell population	proliferating tumor cells (Ki67+CK+)
PD1CD8T	cell population	PD-1 positive cytotoxic T cells (PD-1+ CD8+ CD3+)
PD1CD8nT	cell population	PD-1 positive CD8 negative T cells (PD-1+ CD8- CD3+)
PD1T	cell population	PD-1 positiveT cells (PD-1+ CD3+)
PD1Im	cell population	PD-1 positive immune cells (PD-1+ CK-)
PDL1Mac	cell population	PD-L1 positive macrophages (PD-L1+ CD68+ CK-)
PDL1Im	cell population	PD-L1 positive immune cells (PD-L1+ CK-)
PDL1Tum	cell population	PD-L1 positive tumor cells (PD-L1+ CK+)

CPS	immune score	PDL1 combined positive score: [(PDL1Im + PDL1Tum) / (tumor cell count)] * 100
TPS	immune score	PDL1 tumor proportion score: [(PDL1Tum) / (tumor cell count)] * 100

T_Treg	Morisita-Horn index	colocalization of non-Treg T cells & Treg
T_B	Morisita-Horn index	colocalization of T cells & B cells
Tm_B	Morisita-Horn index	colocalization of tumor cells & B cells
Tm_Treg	Morisita-Horn index	colocalization of tumor cells & Treg
Tm_T	Morisita-Horn index	colocalization of tumor cells & T cells
Tm_TIL	Morisita-Horn index	colocalization of tumor cells & TIL
Tm_CD8nT	Morisita-Horn index	colocalization of tumor cels & CD8- T cells
Tm_Tc	Morisita-Horn index	colocalization of tumor cells & CD8+ cytotoxic T cells
Tm_Mac	Morisita-Horn index	colocalization of tumor cells & macrophages
CD8nT_Mac	Morisita-Horn index	colocalization of CD8- T cells & macrophages
Tc_Mac	Morisita-Horn index	colocalization of CD8+ cytotoxic T cells & macrophages
T_Mac	Morisita-Horn index	colocalization of T cells & macrophages

Biomarker	Type	Description
PD1T_PDL1Tm	Morisita-Horn index	colocalization of PD-1+ T cells & PD-L1+ tumor cells
PD1Tc_PDL1Tm	Morisita-Horn index	colocalization of PD-1+ cytotoxic T cells & PD-L1+ tumor cells
CD8nPD1T_PDL1Tm	Morisita-Horn index	colocalization of CD8- PD-1+ T cells & PD-L1+ tumor cells
PD1T_PDL1Im	Morisita-Horn index	colocalization of PD-1+ T cells & PD-L1+ immune cells
PD1Tc_PDL1Im	Morisita-Horn index	colocalization of PD-1+ cytotoxic T cells & PD-L1+ immune cells
CD8nPD1T_PDL1Im	Morisita-Horn index	colocalization of CD8- PD-1+ T cells & PD-L1+ immune cells
PD1T_PDL1	Morisita-Horn index	colocalization of PD-1+ T cells & any PD-L1+ cell
PD1Tc_PDL1	Morisita-Horn index	colocalization of PD-1+ cytotoxic T cells & any PD-L1+ cell
CD8nPD1T_PDL1	Morisita-Horn index	colocalization of CD8- PD-1+ T cells & any PD-L1+ cell

Tm_B.SPS	Spatial Proximity Score	SPS score for tumor cells with a B cell within 20 um
Tm_T.SPS	Spatial Proximity Score	SPS score for tumor cells with a T cell within 20 um
Tm_Treg.SPS	Spatial Proximity Score	SPS score for tumor cells with a Treg cell within 20 um
T_Treg.SPS	Spatial Proximity Score	SPS score for T cells with a Treg cell within 20 um
T_B.SPS	Spatial Proximity Score	SPS score for T cells with a B cell within 20 um
Tm_CD8nT.SPS	Spatial Proximity Score	SPS score for tumor cells with a CD8- T cell within 20 um
Tm_Tc.SPS	Spatial Proximity Score	SPS score for tumor cells with a cytotoxic T cell within 20 um
Tm_Mac.SPS	Spatial Proximity Score	SPS score for tumor cells with a macrophage within 20 um
T_Mac.SPS	Spatial Proximity Score	SPS score for T cells with a macrophage within 20 um
CD8nT_Mac.SPS	Spatial Proximity Score	SPS score for CD8- T cells with a macrophage within 20 um
Tc_Mac.SPS	Spatial Proximity Score	SPS score for cytotoxic T cells with a macrophage within 20 um
PD1T_PDL1Tm.SPS	Spatial Proximity Score	SPS score for PD-1+ T cells with a PD-L1+ tumor cell within 20 um
PD1Tc_PDL1Tm.SPS	Spatial Proximity Score	SPS score for PD-1+ cytotoxic T cells with a PD-L1+ tumor cell within 20 um
CD8nPD1T_PDL1Tm.SPS	Spatial Proximity Score	SPS score for CD8- PD-1+ T cells with a PD-L1+ tumor cell within 20 um
PD1T_PDL1Im.SPS	Spatial Proximity Score	SPS score for PD-1+ T cells with a PD-L1+ immune cell within 20 um
PD1Tc_PDL1Im.SPS	Spatial Proximity Score	SPS score for PD-1+ cytotoxic T cells with a PD-L1+ immune cell within 20 um
CD8nPD1T_PDL1Im.SPS	Spatial Proximity Score	SPS score for CD8- PD-1+ T cells with a PD-L1+ immune cell within 20 um
PD1T_PDL1.SPS	Spatial Proximity Score	SPS score for PD-1+ T cells with any PD-L1+ cell within 20 um
PD1Tc_PDL1.SPS	Spatial Proximity Score	SPS score for PD-1+ cytotoxic T cells with any PD-L1+ cell within 20 um
CD8nPD1T_PDL1.SPS	Spatial Proximity Score	SPS score for CD8- PD-1+ T cells with any PD-L1+ cell within 20 um
