# Supplementary material for: Multi-platform biomarkers of response to an immune checkpoint inhibitor in the neoadjuvant I-SPY 2 trial for early-stage breast cancer
Source: Cell Rep Med. 2024 Nov 6;5(11):101799. doi: 10.1016/j.xcrm.2024.101799 (PMC11604542; doi:10.1016/j.xcrm.2024.101799)
Supplement: Document S2. Article plus supplemental information [file mmc5.pdf]

Cell Reports Medicine

Article

Multi-platform biomarkers of response to an immune checkpoint inhibitor in the neoadjuvant I-SPY 2 trial for early-stage breast cancer

Graphical abstract

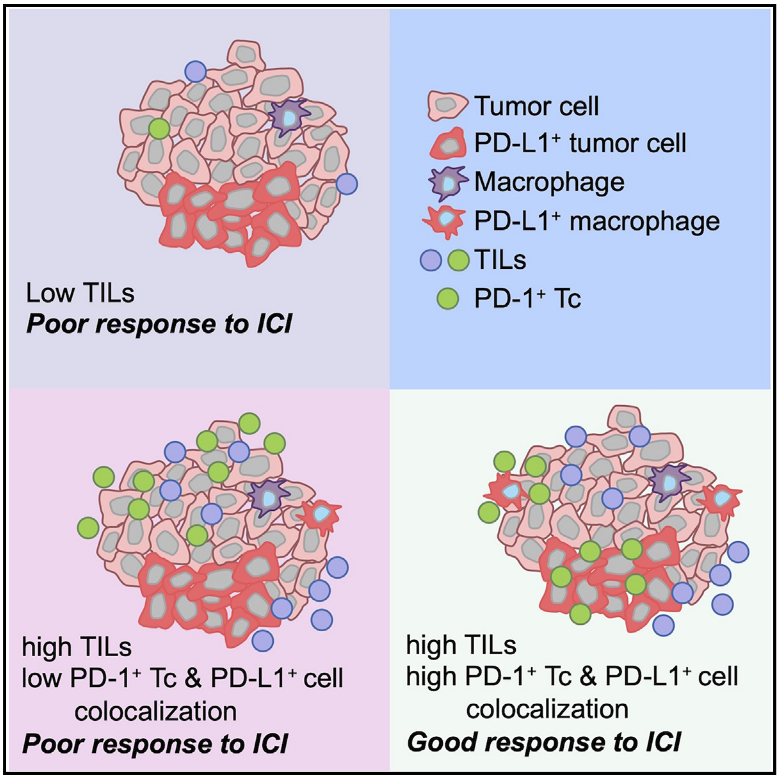

Highlights

- Multiomics analysis of breast cancer treated with neoadjuvant ICB plus chemotherapy
- Immune infiltrates and immune/cytokine signaling pathways correlate with response
- Patterns of response-associated biomarkers differ by receptor subtype
- Tumors with high TILs and PD1/PDL1 colocalization most likely to benefit from ICB

Authors

Michael J. Campbell, Denise M. Wolf, Christina Yau, ..., Emanuel F. Petricoin, Laura van't Veer, Laura Esserman

Correspondence

michael.campbell@ucsf.edu

In brief

Campbell et al. utilize multiplex immunofluorescence staining, gene expression profiling, and reverse phase protein arrays to gain insights into the role of the tumor immune microenvironment during neoadjuvant immunotherapy for breast cancer. Their results suggest that immune cell densities combined with spatial colocalization metrics are relevant for determining clinical outcomes.

Campbell et al., 2024, Cell Reports Medicine 5, 101799
November 19, 2024 © 2024 The Author(s). Published by Elsevier Inc.
https://doi.org/10.1016/j.xcrm.2024.101799

CellPress

Cell Reports Medicine

CellPress OPEN ACCESS

Article

Multi-platform biomarkers of response to an immune checkpoint inhibitor in the neoadjuvant I-SPY 2 trial for early-stage breast cancer

Michael J. Campbell,[1,18,*] Denise M. Wolf,[2] Christina Yau,[1] Lamorna Brown-Swigart,[2] Julie Wulfkuhle,[3] Isela R. Gallagher,[3] Zelos Zhu,[1] Jennifer Bolen,[4] Scott Vandenberg,[5] Clifford Hoyt,[6] Hidetoshi Mori,[7] Alexander Borowsky,[7,8] Laura Sit,[1] Jane Perlmutter,[9] Smita M. Asare,[10] I-SPY2 Investigators, Rita Nanda,[11] Minetta C. Liu,[12] Douglas Yee,[13] Angela M. DeMichele,[14] Nola M. Hylton,[15] Lajos Pusztai,[16] Donald A. Berry,[17] Gillian L. Hirst,[1] Emanuel F. Petricoin,[3] Laura van't Veer,[2] and Laura Esserman[1]

[1]Department of Surgery, University of California, San Francisco, San Francisco, CA 94143, USA
[2]Department of Laboratory Medicine, University of California, San Francisco, San Francisco, CA 94143, USA
[3]Center for Applied Proteomics and Molecular Medicine, George Mason University, Manassas, VA 20110, USA
[4]Biospecimen Resource Program (BIOS), University of California, San Francisco, San Francisco, CA 94143, USA
[5]Department of Pathology, University of California, San Francisco, San Francisco, CA 94143, USA
[6]Akoya Biosciences, Marlborough, MA 01752, USA
[7]Center for Immunology and Infectious Diseases, University of California, Davis, Davis, CA 95616, USA
[8]Department of Pathology and Laboratory Medicine, University of California Davis, Sacramento, CA 95817, USA
[9]Gemini Group, Ann Arbor, MI 48107, USA
[10]Quantum Leap Healthcare Collaborative, San Francisco, CA 94118, USA
[11]Department of Medicine, Section of Hematology/Oncology, University of Chicago, Chicago, IL 60637, USA
[12]Department of Surgery, Mayo Clinic, Rochester, MN 55905, USA
[13]Department of Medicine, University of Minnesota, Minneapolis, MN 55455, USA
[14]Perelman School of Medicine, University of Pennsylvania, Philadelphia, PA 19104, USA
[15]Department of Radiology, University of California, San Francisco, San Francisco, CA 94143, USA
[16]Yale School of Medicine, Yale University, New Haven, CT 06510, USA
[17]Berry Consultants, LLC, Austin, TX 78746, USA
[18]Lead contact
*Correspondence: michael.campbell@ucsf.edu
https://doi.org/10.1016/j.xcrm.2024.101799

SUMMARY

Only a subset of patients with breast cancer responds to immune checkpoint blockade (ICB). To better understand the underlying mechanisms, we analyze pretreatment biopsies from patients in the I-SPY 2 trial who receive neoadjuvant ICB using multiple platforms to profile the tumor microenvironment. A variety of immune cell populations and markers of immune/cytokine signaling associate with pathologic complete response (pCR). Interestingly, these differ by breast cancer receptor subtype. Measures of the spatial distributions of immune cells within the tumor microenvironment, in particular colocalization or close spatial proximity of PD-1^{+} T cells with PD-L1^{+} cells (immune and tumor cells), are significantly associated with response in the overall cohort as well as the in the triple negative (TN) and HR^{+}HER2^{-} subtypes. Our findings indicate that biomarkers associated with immune cell signaling, immune cell densities, and spatial metrics are predictive of neoadjuvant ICB efficacy in breast cancer.

INTRODUCTION

Immunotherapy with immune checkpoint inhibitors (ICIs) has changed the landscape of cancer treatment. In various tumor types, immune checkpoint blockade leads to durable responses, albeit in only a subset of patients.[1–4] In breast cancer, there has been tremendous progress over the past few years with several trials reporting promising efficacy of ICIs in both the adjuvant and neoadjuvant setting. Five randomized trials have reported results from neoadjuvant therapy with ICIs in breast cancer (KEYNOTE-522, NeoTRIPaPDL1, GeparNuevo, Impassion031, and I-SPY 2) with pathologic complete response (pCR) rates in triple-negative breast cancer (TN) ranging from 53% to 65%.[5–9]

As has been seen in other tumor types, only a subset of patients with breast cancer responds to ICIs in either the adjuvant or neoadjuvant setting. Biomarkers are being developed and evaluated to predict responsiveness to ICIs. Given the potential for long-term side effects from ICIs, it is important to identify which early-stage breast cancers will be the most likely to respond, or not. Initial research across various cancer types focused on characterizing the tumor immune microenvironment and classifying tumors as immune "hot,"

This is an open access article under the CC BY license (http://creativecommons.org/licenses/by/4.0/).

"cold," or "excluded."[10] Immune "hot" tumors are presumed to be primed for an immune response and more likely to respond to ICIs compared to immune "cold" or "excluded." Gene expression signatures (GESs) have been used to characterize the tumor immune microenvironment, and several have been shown to correlate with immune infiltration and activity and to predict for tumors more likely to respond to ICIs.[11] In the GeparNuevo trial (neoadjuvant durvalumab + chemotherapy), gene expression profiling identified several genes associated with a treatment interaction, suggesting they may be useful biomarkers for future studies.[12] Intrinsic properties of the tumor itself, such as tumor mutational burden, may also point to patients who are more likely to respond to immune checkpoint blockade. However, this association is only observed in some tumors.[13]

PD-L1 immunohistochemistry (IHC) has been used as a biomarker for immune checkpoint blockade. Indeed, PD-L1 IHC with the 22C3 antibody was the first US Food and Drug Administration (FDA)-approved biomarker for use with pembrolizumab for non-small cell lung cancer (NSCLC).[14] However, PD-L1 IHC of tumor and/or immune cells has produced inconsistent results and does not always discriminate responders from non-responders to ICIs.[15,16] For neoadjuvant therapy with ICIs in breast cancer, PD-L1 expression by IHC did not predict selective benefit from inclusion of ICIs with chemotherapy in either the KEYNOTE-522 or the NeoTRIPaPDL1 trials.[5,7] PD-1 and PD-L1 staining can be technically difficult to interpret.[17] In one study of 68 breast cancers stained with the SP142 anti-PD-L1 antibody, only 38% of cases had complete concordance among 19 pathologists.[18] Another study found low overall concordance among three different IHC assays in identifying PD-L1-positive cases.[19]

Several technologies that facilitate the assessment of multiple markers while preserving the spatial relationships of cells *in situ* have been developed in recent years.[20] These methodologies are being used to characterize the tumor immune microenvironment and for immune biomarker discovery. A recent report has shown that multiplexed immunofluorescence (mIF) assays can outperform GESs, tumor mutational burden, and PD-L1 expression (measured by standard IHC) for predicting response to checkpoint inhibition across several tumor types[21]

The I-SPY 2 trial is a multicenter phase 2 adaptive standing platform trial for women with early-stage, locally advanced, aggressive breast cancer. I-SPY 2 is a biomarker-rich trial that collects tumor tissue pretreatment for multi-platform molecular and spatial profiling. In this study, we explored the densities and spatial distributions of immune cells within the tumor microenvironment of breast cancers from patients treated with neoadjuvant chemotherapy (control arm) or neoadjuvant chemotherapy plus pembrolizumab (Pembro arm) in the I-SPY 2 trial using mIF. These mIF measures were assessed as specific predictors of response to pembrolizumab. Immune signaling and DNA repair deficiency biomarkers and associated signaling pathways were also assessed using gene expression and reverse phase protein arrays (RPPAs).

RESULTS

Biomarker study population

A total of 69 $HER2^-$ patients (40 HR^+HER2^- and 29 TN) were randomized to receive 4 cycles of pembrolizumab in combination with weekly paclitaxel followed by anthracycline chemotherapy (Pembro+T → AC). In addition, there were 181 $HER2^-$ patients (96 HR^+HER2^- and 85 TN) randomized to the standard neoadjuvant chemotherapy control group (T → AC). We utilized three assay platforms to characterize the tumor immune microenvironment in these patients (Figure 1). Of the 250 cases, 99% ($n = 248$) had Agilent 44K expression array data available and 94% ($n = 236$) had available RPPA data from laser-microdissected tumor epithelium. Fifty-four of the 69 patients on the Pembro arm were evaluated by mIF.

Thirty-eight expression signatures were evaluated from the expression array data. These included 26 immune-related signatures, 10 DNA damage response (DDR) signatures, 1 proliferation signature, and 1 hormone receptor (estrogen receptor/progesterone receptor; ER/PR) signature (see Table S2 for genes and references). The RPPA data contained 17 immune-related and 10 DDR-related protein/phosphoprotein markers (Table S3). Finally, the mIF analyses yielded 61 biomarkers: 18 cell populations, 2 PD-L1 scores (combined positive score [CPS] and tumor proportion score [TPS]), and 41 spatial metrics (21 based on the Morisita-Horn (MH) index and 20 based on the nearest-neighbor distribution function) (Table S4). Overall, this resulted in 126 biomarkers per case.

Identification of immune cell infiltrates in breast cancer biopsies by mIF

Two mIF panels were developed to identify immune cell infiltrates in pretreatment biopsies (see Table S1 for the markers and antibodies used in each panel). The first panel was designed to identify cytokeratin$^+$ ($CK^{+)}$ tumor/epithelial cells, $CD3^+$ T cells, $CD3^+Foxp3^+$ regulatory T cells (Tregs), $CD20^+$ B cells, and $CD117^+CK^-$ mast cells (Figure 2A). The combination of T cell and B cell counts was used to define tumor-infiltrating lymphocytes (TIL). In addition, proliferating tumor cells, T cells, and B cells were identified based on their staining with Ki67 in this panel. The second panel identified CK^+ tumor/epithelial cells, $CD8^+$ cytotoxic T cells (Tc), $CD8^-$ T cells, and $CD68^+$ macrophages, as well as the expression of PD-1 and PD-L1 on these cells (Figure 2B). Phenotyping of these cell populations was achieved using auto-gating algorithms for each marker as shown in Figures 2C and 2D. Phenotype maps, generated for the images shown in Figures 2A and 2B, are shown in Figures 2E and 2F. These were used for subsequent spatial analyses.

Immune cell densities varied widely across tumors (Figure 2G). The fraction of immune cells (T cells + B cells + macrophages) ranged from 1% to 68% of total cells. This variability was also observed for each cell type (T cells: <1%–42%; B cells: <1%–22%; macrophages: <1%–43%) (Figure 2G). There were significant correlations between T cell, B cell, and Treg densities (Figure 2H); however, macrophage and mast cell densities did not correlate with these lymphoid cell populations. PD-1 and PD-L1 staining also varied widely across tumors (Figure 2I). The fraction of PD-1$^+$ T cells ranged from <0.04% to 3.9% of total cells, and the fraction of PD-L1$^+$ cells (immune and tumor cells) ranged from <0.06% to 2.6% of total cells.

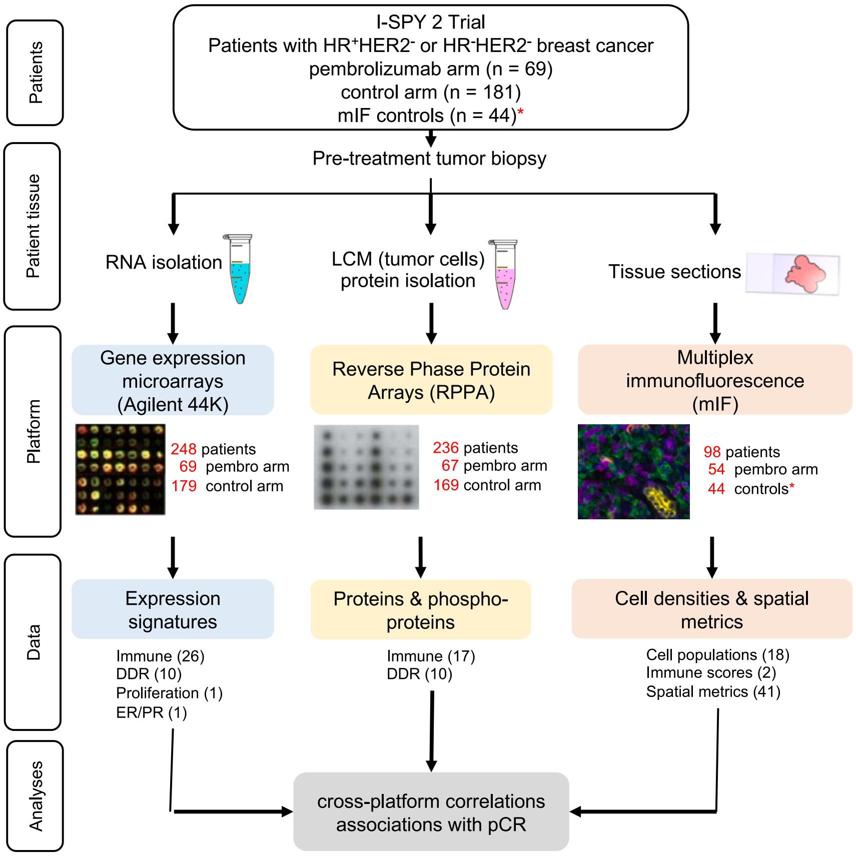

Figure 1. Overview of tissue collection and analyses

Pretreatment tumor biopsies were processed for biomarker evaluation on three different platforms: gene expression microarrays, reverse phase protein arrays, and multiplex immunofluorescence. The number of samples analyzed under each platform is shown in red. 38 expression signatures (26 immune, 10 DDR, 1 proliferation, and 1 ER/PR), 27 RPPA biomarkers (17 immune and 10 DDR), and 61 mIF biomarkers (18 cell populations, 2 immune scores, and 41 spatial metrics) were evaluated. *The 44 control tissue samples acquired for mIF analyses were not from the concurrent control arm (see STAR Methods for details).

Cross-platform correlation of several immune markers

Data were available from both mIF and expression array analyses for ten cross-platform comparisons. Significant correlations were observed for 7/10 of these comparisons (range of correlations: −0.01 to 0.65) (Figures S1A–S1J). These included a T cell GES and T cells measured by mIF (Figure S1A) as well as a CD8^{+} T cell GES and CD8^{+} T cells by mIF (Figure S1B). Ki67^{+} proliferating T cells (mIF) correlated with an activated T cell gene signature (TcClassII_sig) (Figure S1C), and, interestingly, PD-1^{+} T cells (mIF) were significantly associated with an exhausted T cell gene signature (which did not contain the gene for PD-1; PDCD1) (Figure S1D). B cells and TILs also correlated across the mIF and expression array platforms (Figures S1E and S1F, respectively). In addition, TIL counts by mIF were correlated with a T&B cell gene signature (Module4 T&B) (Figure S1G). In contrast, measurement of Tregs, macrophages, and mast cells by mIF did not correlate with their respective gene signatures (Figures S1H–S1J, respectively). Finally, we examined two cross-platform correlations between the RPPA and mIF platforms. The T cell-related RPPA markers, CD3ε and CD3ζ, did not correlate with T cell infiltrates measured by mIF (Figures S1K and S1L).

We compared 12 PD-1 and PD-L1 markers across all three platforms and found 23 significant correlations among these markers (Figure S1M). mIF identification of PD-1^{+} T cells (PD1T) significantly correlated with expression of the PD-1 gene (PDCD1), but not with RPPA results from two anti-PD-1 antibodies (Pembro and nivolumab [Nivo]). Since RPPA was performed on laser microdissected epithelial cell regions, not stroma, and PD-1^{+} T cells identified by mIF were predominantly located in the stroma, this could account for the lack of correlation. While the fraction of PD-L1^{+} tumor cells (PDL1Tum) and PD-L1^{+} immune cells (PDL1Imm) measured by mIF with the E1L3N anti-PD-L1 antibody correlated with each other, neither cell population was significantly correlated with expression of the PD-L1 gene (CD274), nor with RPPA results from several anti-PD-L1 antibodies (22C3, SP142, E1L3N, 28.8, atezolizumab [Atezo]) (Figure S1M). Interestingly, among the PD-L1 RPPA data, there was variable correlation between the different anti-PD-L1 clones, and only E1L3N correlated with CD274 gene expression.

Numerous immune biomarkers were associated with response to neoadjuvant immunotherapy

We examined the association of immune cell population markers with response (pCR) in both the Pembro arm and the control arm, in all cases and by receptor subtypes. Figure 3 shows those markers that were significantly associated with pCR (after Benjamini-Hochberg multiple testing correction) in at least one of the cohorts. In general, levels of these markers were positively correlated with each other with the exception of Tregs, PD-L1^{+} immune cells, PD-L1^{+} macrophages, the PD-L1 CPS, and the mast cell signature (Figure S2).

Of the 20 immune cell populations or scores identified by mIF (Table S4), 8 were positively associated ($p < 0.05$, Benjamini-Hochberg (BH) corrected) with pCR in the Pembro arm (Figures 3 and S3). These included TILs, total T cells, CD8^{-} T cells, Tregs, proliferating T cells, two PD-L1+ populations, and the PD-L1 CPS. TIL, T cells, and B cells were also associated with pCR in the control arm, although less significantly (Figure 3A, $p < 0.05$, uncorrected). Eighteen GESs/markers were used to characterize immune infiltrates (Table S2), and 11 of these were associated with response to therapy in the Pembro arm but not the control arm

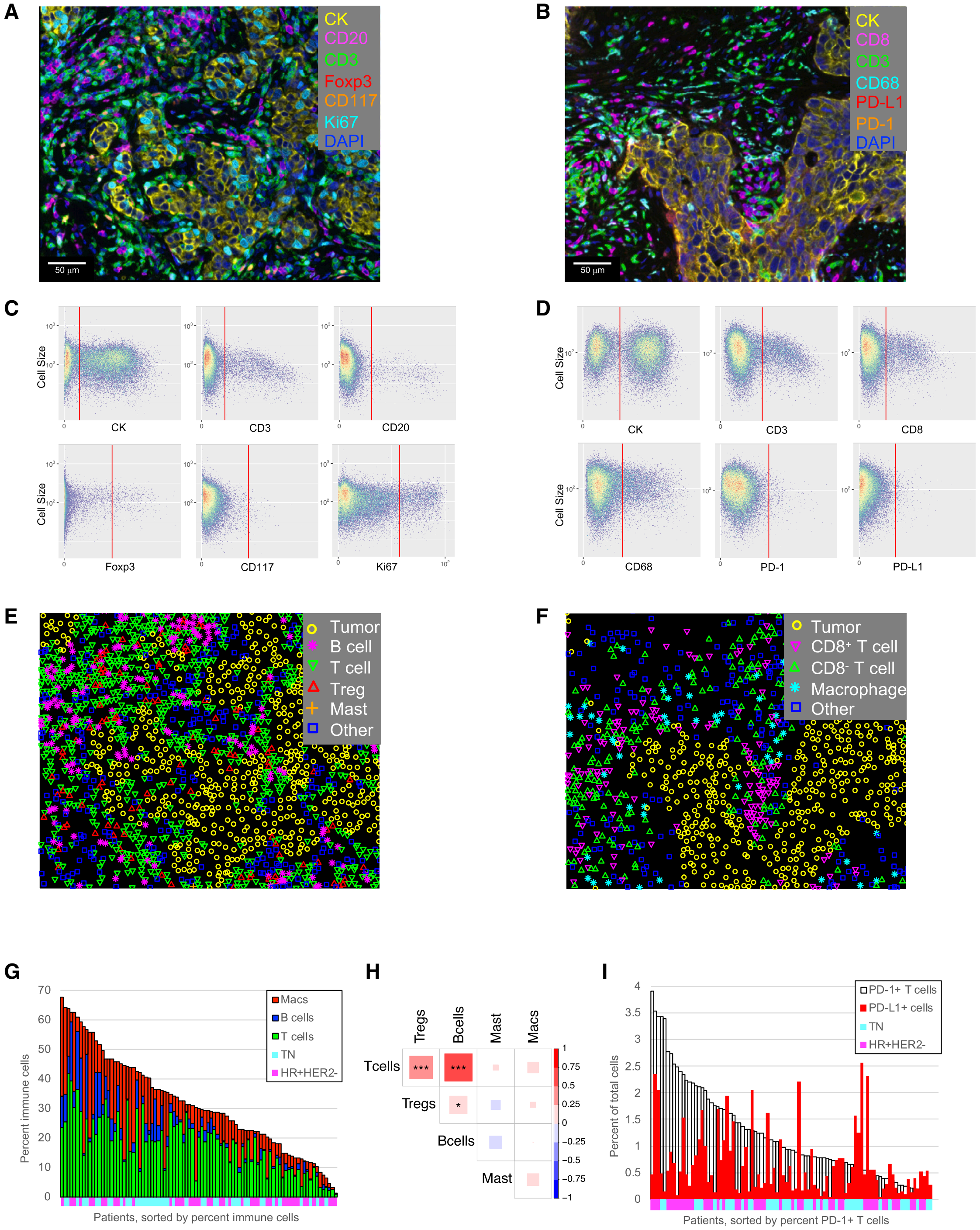

Figure 2. mIF analysis of immune cells in the breast cancer microenvironment

(A) Example breast cancer tissue stained with mIF panel 1. Cells are pseudo-colored as indicated for the different markers.
(B) Example breast cancer tissue stained with mIF panel 2. Cells are pseudo-colored as indicated for the different markers.
(C) Example of auto-generated gates for each marker in mIF staining panel 1.
(D) Example of auto-generated gates for each marker in mIF staining panel 2.
(E) Phenotype map corresponding to the image in (A).
(F) Phenotype map corresponding to the image in (B).
(G) Immune cell densities across the entire cohort (*n* = 98 patients). Patients sorted by percentage of immune cells, colored by cell phenotype. Annotations along the x axis indicate receptor subtype (cyan: TN; magenta: $HR^{+}HER2^{-}$).

(legend continued on next page)

(Figures 3 and S3). These included gene signatures for various immune cell types (cytotoxic cells, exhausted T cells, Th1 cells, B cells, natural killer cells, dendritic cells [DCs], and mast cells), a T and B cell signature (Mod4_TB), a Tc/major histocompatibility complex (MHC) class II signature (TcClassII_sig), and the genes for PD-1 and PD-L1 (PDCD1 and CD274, respectively). Finally, we evaluated 11 immune cell surface markers by RPPA, two T cell markers (CD3ε and CD3ζ), two PD-1 markers, five PD-L1 markers, and two MHC classII markers (Table S3). Of these, only one of the MHC class II markers (HLADRPQX), which detects HLA-DR, HLA-DP, HLA-DQ, and HLA-DX, was associated with response in the Pembro arm but not the control arm (Figures 3 and S3). Overall, 20/49 immune cell markers were significantly associated ($p < 0.05$, BH corrected) with response to Pembro in the whole cohort, while 6 markers were nominally associated ($p < 0.05$, not corrected) with response in the control arm. The DC signature (DC_sig) showed the strongest positive association with pCR, and the mast cell signature (Mast_sig) showed the strongest negative association.

The immune biomarkers associated with pCR differed by receptor subtypes. In TN tumors, 3 markers were significantly associated with response to Pembro: PDL1$^+$ immune cells measured by mIF (PDL1Im), a Th1 gene signature (Th1_sig), and a DC gene signature (DC_sig), while an additional 8 markers were nominally associated with response in this subtype (Figure 3A). In contrast, 10 markers were significantly associated with response to Pembro in HR$^+$HER2$^-$ tumors, with an additional 9 nominally significant markers. One of these was HLADRPQX measured on the RPPA platform, and the other nine were immune cell populations identified by mIF including TILs, T cells, CD8$^-$ T cells, Tregs, B cells, proliferating B cells, and three populations of PD-1$^+$ cells (Figure 3A). None of these markers were associated with response in the control arm within either subtype.

Finally, adjusting for various clinical variables (age, race, longest diameter by MRI at baseline, and palpable nodes [yes/no]) did not result in the loss of any significant mIF-based biomarkers (data not shown). Adjusting for these variables resulted in loss of only 2 significant GESs (ExhTc_sig and NK56d_sig), although these remained significant without BH correction (data not shown).

Colocalization of immune cells and tumor cells associated with response to pembrolizumab

We investigated whether the proximity of various immune cell populations to tumor cells or other immune cells differed between responders and non-responders. We utilized two spatial proximity measures, the MH index and the nearest-neighbor distribution function, for these analyses. The MH index gives a measure of the degree of colocalization of two cell types. As depicted in Figure 4A, when two cell types (TIL and cancer cells in this example) are highly colocalized, the MH index (which ranges from 0 to 1) will be near 1. In contrast, highly segregated (low colocalization) cell populations will generate an MH index value close to 0. The MH index was calculated for 21 pairs of cell types (Table S4). An example of high colocalization of T cells and Tregs is shown in Figure 4B. High T_Treg MH index values were significantly associated with response to Pembro in the whole cohort (Figure 4C). Figure 4D illustrates a case with high colocalization of PD-1$^+$ T cells and any PD-L1$^+$ cell (tumor or immune), and the associated MH index (PD1T_PDL1) was also associated with pCR to Pembro (Figure 4E).

Of the 21 MH indices evaluated (Table S4), 8 were associated with response to Pembro in the overall cohort, after correcting for multiple testing, but not in the controls (Figures 4F and S4). Six of these were related to PD-1/PD-L1 colocalizations, and 3 demonstrated a significant interaction with treatment (PD1Tc_PDL1, PD1T_PDL1Tm, and PD1Tc_PDL1Tm).

Seven MH indices were positively associated with pCR in the Pembro arm but not the control arm when evaluated within receptor subtypes (Figure 4F). All of these were related to PD-1/PD-L1 colocalizations and were highly correlated with each other (Figure S2). In particular, colocalizations of PD-1$^+$ T cells with any PD-L1$^+$ cell or with PD-L1$^+$ tumor cells, as well as colocalization of PD-1$^+$ CD8$^+$ Tc cells with any PD-L1$^+$ cell, were associated with pCR in both TN and HR$^+$HER2$^-$ tumors.

Spatial proximity of immune cells and tumor cells associated with response to pembrolizumab

To further characterize the spatial relationships between cells in the tumor microenvironment, we utilized the nearest-neighbor distribution function, $G(r)$, to evaluate the probability of a cell of type "*a*" having at least one cell of type "*b*" within a distance r. As illustrated in Figure 5A, the area under the $G(r)$ function curve, from 0 to 20 μm, was used to generate a spatial proximity score (SPS) for a given pair of cell types. Figure 5B and 5C illustrate SPSs calculated for tumors with a low and a high tumor-to-T cell proximity score (Tm_T.SPS), respectively. A high Tm_T.SPS value indicates that a high fraction of tumor cells have T cells nearby. High Tm_T.SPS values were significantly associated with response to Pembro (Figure 5D). Figures 5E and 5F illustrate SPSs calculated for tumors with low and high proximities of PD-1+T cells with PD-L1+ cells (tumor or immune). A high PD1T_PDL1.SPS value indicates that a high fraction of PD-1$^+$ T cells have a PD-L1$^+$ cell nearby. Significantly higher PD1T_PDL1.SPS values were associated with pCR (Figure 5G).

SPSs were determined for 20 pairs of cell types (Table S4). Eight of these scores were associated with response to Pembro in the overall cohort, after correcting for multiple testing, but not in the control arm (Figures 5H and S5). Three of these were related to tumor/lymphocyte proximities (Tm_T.SPS, Tm_CD8nT.SPS, Tm_Treg.SPS) and 5 were related to PD-1/PD-L1 proximities (PD1T_PDL1.SPS, PD1T_PDL1Im.SPS, PD1Tc_PDL1.SPS, PD1Tc_PDL1Im.SPS, CD8nPD1T_PDL1Im.SPS).

(H) Correlation of immune cell populations across patients (n = 98 patients). Pearson correlations, red indicates positive correlation, blue indicates negative correlation. *$p < 0.05$, **$p < 0.01$, ***$p < 0.001$.

(I) Association of PD-1$^+$ T cell density (white bars) with PD-L1$^+$ cell density (red bars). Patients sorted by percentage of PD-1$^+$ T cells (n = 98 patients). Annotations along the x axis indicate receptor subtype (cyan: TN; magenta: HR$^+$HER2$^-$).

See also Table S1.

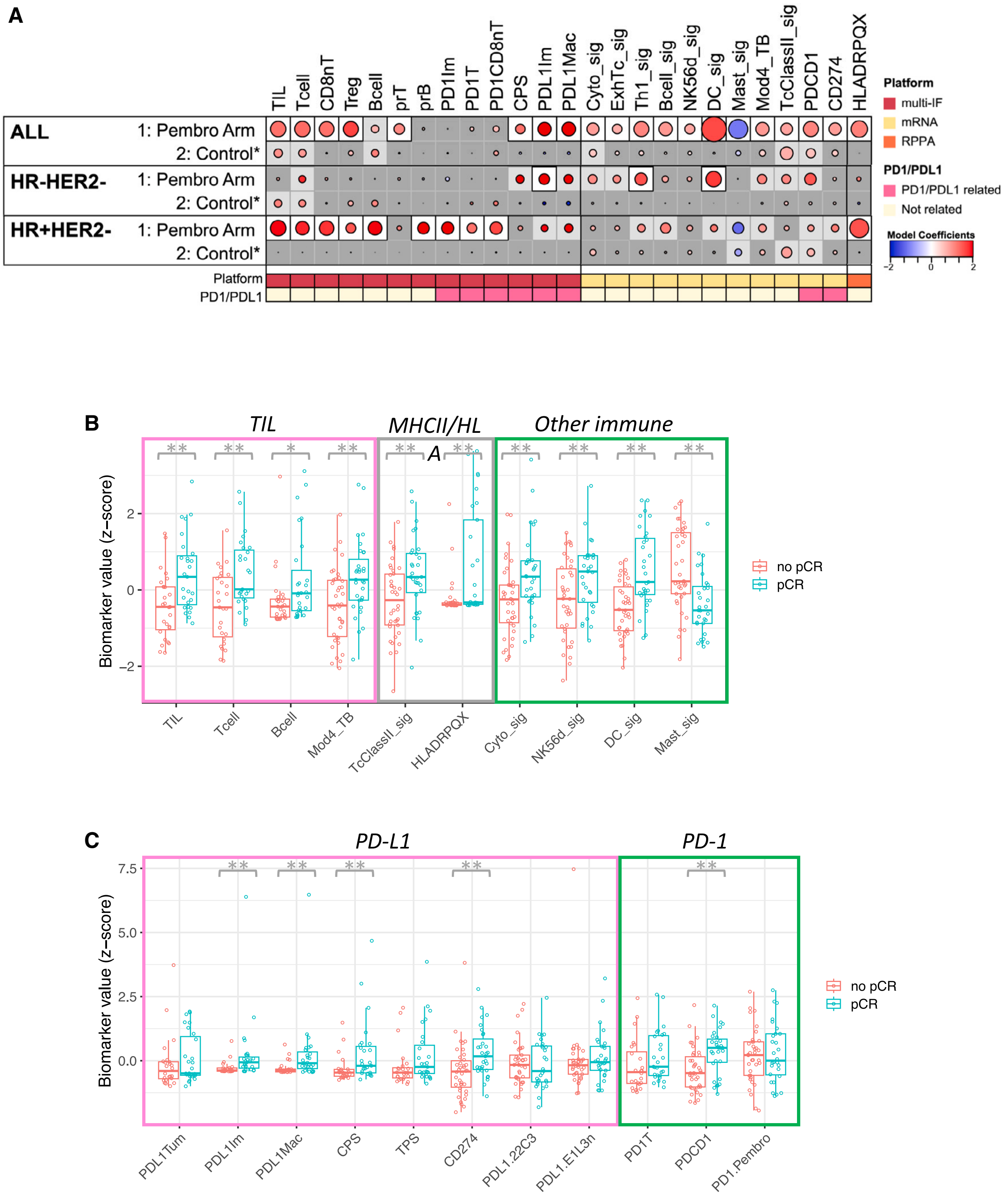

Figure 3. Immune biomarkers associated with response to ICB

(A) Association dot matrix showing the level and direction of association between each immune predictive biomarker (columns) and pCR in the population/model as labeled (rows). Only those biomarkers that were significant ($p < 0.05$; after Benjamini-Hochberg multiple testing correction) in at least one cohort are shown. ALL, Pembro Arm, multi-IF platform ($n = 54$ patients); ALL, Pembro Arm, mRNA platform ($n = 69$ patients); ALL, Pembro Arm, RPPA platform ($n = 67$ patients); ALL, Control, multi-IF platform ($n = 44$ patients); ALL, Control, mRNA platform ($n = 179$ patients); ALL, Control, RPPA platform ($n = 169$ patients); HR−HER2−, Pembro Arm, multi-IF platform ($n = 24$ patients); HR−HER2−, Pembro Arm, mRNA platform ($n = 29$ patients); HR−HER2−, Pembro Arm, RPPA platform ($n = 27$ patients); HR−HER2−, Control, multi-IF platform ($n = 23$ patients); HR−HER2−, Control, mRNA platform ($n = 85$ patients); HR−HER2−, Control, RPPA platform ($n = 78$ patients); HR+ HER2−, Pembro Arm, multi-IF platform ($n = 30$ patients); HR+ HER2, Pembro Arm, mRNA platform ($n = 40$ patients); HR+ HER2−, Pembro Arm, RPPA platform ($n = 40$ patients); HR+ HER2−, Control, multi-IF platform ($n = 21$ patients); HR+ HER2−, Control, mRNA platform ($n = 94$ patients); HR+ HER2−, Control, RPPA platform ($n = 91$ patients). Color of dot indicates direction of association (red, higher in pCR; blue, higher in non-pCR). Size of dot is proportional to significance (larger dots → smaller p values). Background square color indicates BH false discovery rate [FDR] $p < 0.05$ (white), nominal $p < 0.05$ (light gray), not significant (dark gray).

(legend continued on next page)

CellPress
OPEN ACCESS

Three SPSs, all related to PD-1/PD-L1 proximities, were associated with pCR in TN tumors (PD1Tc_PDL1.SPS, PD1Tc_PDL1Tm.SPS, CD8nPD1T_PDL1Im.SPS). In $HR^{+}HER2^{-}$ tumors, 2 PD-1/PD-L1-related SPSs (PD1Tc_PDL1.SPS, PD1Tc_PDL1Im.SPS) and 3 other SPSs (Tm_Treg.SPS, Tm_B.SPS, T_B.SPS) were significantly associated with response to Pembro (Figure 5H).

Immune signaling pathways associated with response to pembrolizumab

We evaluated 8 expression-based and 6 RPPA-based biomarkers related to immune signaling pathways, as well as a hormone receptor gene signature and a proliferation gene signature (Tables S2 and S3), for their associations with response to pembrolizumab immunotherapy. Of these 16 biomarkers, 9 were significantly associated with pCR in the Pembro arm, but not the control arm (Figures 6 and S6; corrected for multiple testing). These included a chemokine signature (Chemokine12), the GeparSixto signature (a set of 12 immune genes that predicted response to neoadjuvant chemotherapy in breast cancer in the GeparSixto trial), an integrated cytokine score gene signature (ICS5), a tumor inflammation signature (TIS; a set of 18 genes that measures a pre-existing, suppressed adaptive immune response), a STAT1 gene signature, STAT1 and STAT3 phosphoprotein biomarkers, a hormone receptor expression gene signature (ER_PR_sig), and a proliferation signature (Mitotic_sig). The Chemokine12 and STAT1 expression signatures showed the highest positive association with response, while the ER_PR expression signature showed the greatest negative association with response. When evaluated by receptor subtypes, the Chemokine12, TIS, and STAT1 signatures were associated with pCR in the Pembro arm in TN tumors whereas none of these markers were associated with response to Pembro in the $HR^{+}HER2^{-}$ cohort (Figure 6). Interestingly, in the $HR^{+}HER2^{-}$ cohort, the Chemokine12 expression signature and phospho-STAT1 RPPA biomarkers were associated with response in the control arm but not the Pembro arm.

DNA damage and repair biomarkers associated with response to pembrolizumab

Given that pembrolizumab has been approved for treatment of microsatellite instability-high and mismatch repair-deficient cancers, we were interested in assessing DNA damage and repair deficiency biomarkers as predictors of sensitivity to this agent. We tested 10 GESs and 10 proteins/phosphoproteins reflecting different aspects of DNA damage, deficiency, and repair. Expression-based biomarkers included PARPi7/MP2, a signature that predicted sensitivity to veliparib/carboplatin in I-SPY 2, and Fanconi anemia (FA), mismatch repair (MMR), base excision repair (BER), homologous recombination (HR), translesion synthesis (TLS), nucleotide excision repair (NER), non-homologous end joining (NHEJ), direct repair (DR), and DNA damage sensing (DamageSens) gene expression signatures (Table S2). On the protein/phosphoprotein level, we tested DNA repair deficiency markers pBRCA1, pCHK1, pCHK2, and pPARP-cleaved; apoptosis/DNA damage markers pCaspase-3-cleaved, pCaspase-9-cleaved, and pH2AX; and microsatellite instability markers MLH1, MSH6, and MSH2, all assayed using RPPA (Table S3). Of the 20 biomarkers related to DNA damage, deficiency, and repair, only the DamageSens GES was associated with pCR in the Pembro arm, but not the control arm (Figures 6 and S7).

Immune microenvironments associated with response to pembrolizumab

As can be seen in Figures 3, 4, 5, and 6, the associations of biomarkers with response differed between receptor subtypes, suggesting distinct response mechanisms. $HR^{+}HER2^{-}$ tumors tended to have fewer TILs (T + B cells measured by mIF) compared to TN tumors. In this study, 29% (8/28) of $HR^{+}HER2^{-}$ tumors were TIL low (<12.5% of total cells) whereas only 4% (1/23) of TN tumors were TIL low (Figure 7A). Irrespective of subtype, tumors with low TILs did not respond to pembrolizumab (0% pCR rates; Figure 7A). In contrast, tumors with high TILs demonstrated higher pCR rates, and these differed by subtype: 82% in TN vs. 45% in $HR^{+}HER2^{-}$. Tumors with high TILs can be further characterized as having high or low colocalization of $PD\text{-}1^{+}$ Tc with $PD\text{-}L1^{+}$ cells (PD1Tc_PDL1-MH score). Thirty-five percent (7/20) of $HR^{+}HER2^{-}$ tumors with high TILs had low PD1Tc_PDL1-MH scores with a 0% pCR rate. In contrast, the pCR rate for TIL-high $HR^{+}HER2^{-}$ tumors with high PD1Tc_PDL1-MH scores was 69% compared to 45% for the $HR^{+}HER2^{-}$ TIL-high-only group. Taking into account the PD1Tc_PDL1 spatial proximity in the TN tumors, pCR rates also increased, from 82% (TN TIL-high group) to 100% (TN TIL-high/PD1Tc_PDL1-high) (Figure 7A). These results indicate that within the receptor subtypes there are differences in the immune microenvironments related to response to immune checkpoint blockade. As shown in Figure 7B, immune microenvironments defined by percent TILs and colocalization of $PD\text{-}1^{+}$ Tc with any $PD\text{-}L1^{+}$ cell were significantly associated with response to pembrolizumab.

We have recently reported an immune response predictive subtype (RPS) based on GESs where $Immune^{+}$ tumors have a higher likelihood of responding to immunotherapy.[22] In the current study, eight of the nine TIL-low tumors and 11/18 TIL-high/PD1Tc_PDL1-low tumors were classified as RPS-$Immune^{-}$. In contrast, 20/24 TIL-high/PD1Tc_PDL1-high tumors were classified as RPS-$Immune^{+}$. As shown in Figure 7C,

(B) Boxplots illustrating the associations of various biomarkers related to TILs, MHC class II, and other immune cell types with pCR in the Pembro arm. The data are depicted as individual dots for each sample, along with the median, first, and third quartile. Statistical analysis was performed using the likelihood-ratio test. **p* < 0.05 (not corrected), ***p* < 0.05 (BH corrected).

(C) Boxplots illustrating the associations of various biomarkers related to PD-L1 and PD-1 expression with pCR in the Pembro arm. The data are depicted as individual dots for each sample, along with the median, first, and third quartile. Statistical analysis was performed using the likelihood-ratio test. ***p* < 0.05 (BH corrected).

See also Figure S3 and Tables S2, S3, S4, S5, and S6.

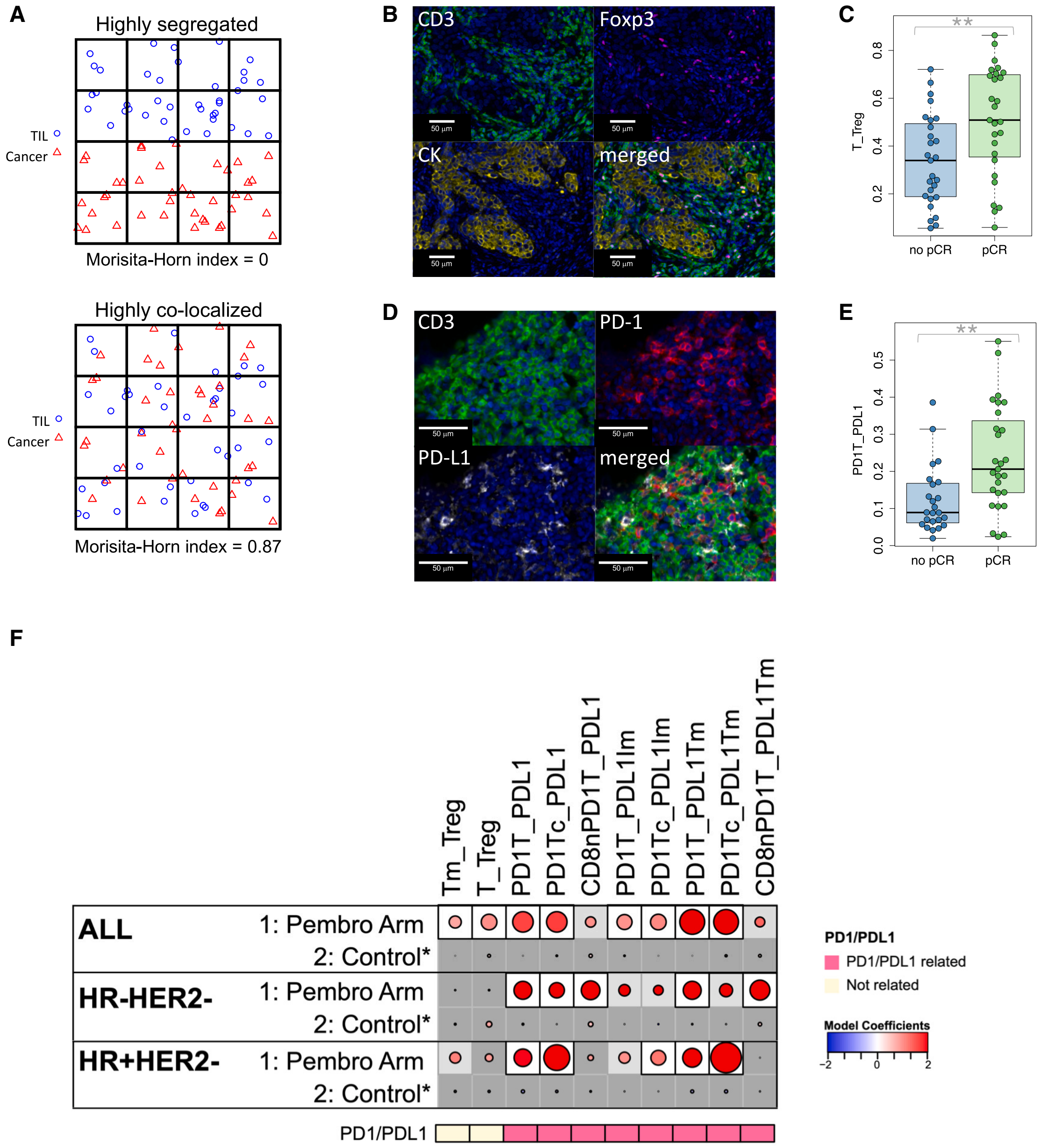

Figure 4. Colocalization of cells in the tumor microenvironment is associated with response to ICB

(A) Schematic representation of a tumor in which TILs are highly segregated away from tumor cells, yielding a low MH index (upper), and a tumor in which TILs are highly colocalized with tumor cells, yielding a high MH index (lower).

(B) Example images from a tumor with high colocalization of $CD3^{+}Foxp3^{-}$ T cells and $CD3^{+}Foxp3^{+}$ Tregs.

(C) Boxplot illustrating significantly higher MH index scores for colocalization of T cells with Tregs (T_Treg) in patients who achieved a pCR. The data are depicted as individual dots for each sample, along with the median, first, and third quartile (n = 54 patients). Statistical analysis was performed using the likelihood-ratio test. $**p < 0.05$ (BH corrected).

(D) Example images from a tumor with high colocalization of $CD3^{+}PD\text{-}1^{+}$ T cells and $PD\text{-}L1^{+}$ cells.

(E) Boxplot illustrating significantly higher MH index scores for colocalization of $PD\text{-}1^{+}$ T cells with any $PD\text{-}L1^{+}$ cell (PD1T_PDL1) in patients who achieved a pCR. The data are depicted as individual dots for each sample, along with the median, first, and third quartile (n = 54 patients). Statistical analysis was performed using the likelihood-ratio test. $**p < 0.05$ (BH corrected).

(F) Association dot matrix showing the level and direction of association between each MH index (columns) and pCR in the population/model as labeled (rows). Only those biomarkers that were significant ($p < 0.05$; after Benjamini-Hochberg multiple testing correction) in at least one cohort are shown. See Figure 3 legend for number of patients in each cohort (multi-IF platform). Color of dot indicates direction of association (red, higher in pCR; blue, higher in non-pCR). Size of dot is proportional to significance (larger dots → smaller p values). Background square color indicates: BH FDR $p < 0.05$ (white), nominal $p < 0.05$ (light gray), not significant (dark gray). See also Figure S4 and Tables S4 and S5.

CellPress
OPEN ACCESS

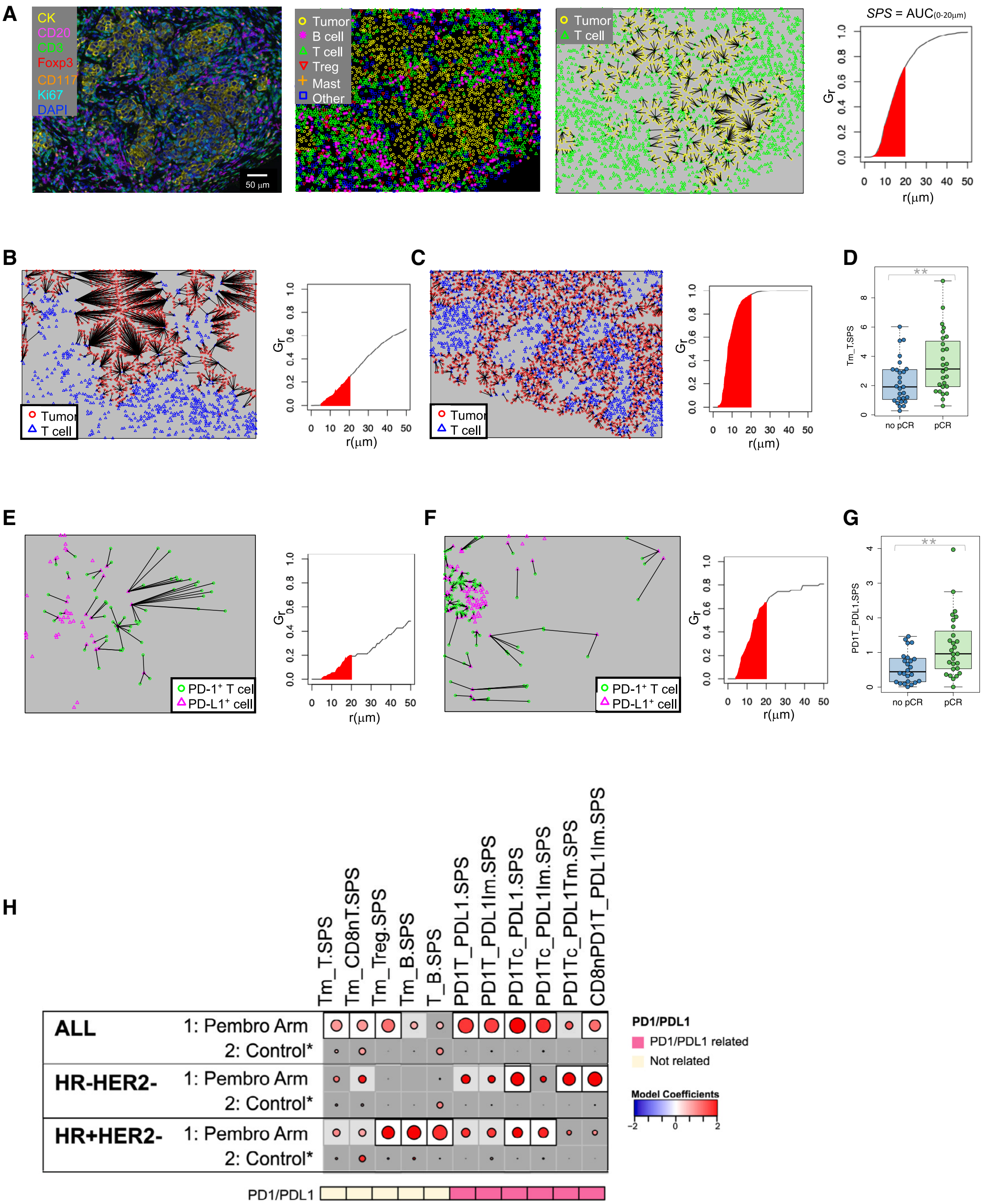

Figure 5. Spatial proximity of cells in the tumor microenvironment is associated with response to ICB

(A) Example of a tumor stained with mIF panel 1, the corresponding phenotype map, and a nearest-neighbor plot in which lines are drawn from each tumor cell (yellow) to the nearest T cell (green). A plot of *G(r)*, the fraction of tumor cells with a T cell with a radius r, by r (μm) is shown. A spatial proximity score (SPS) is calculated from the area under this curve where r = 0 to 20 μm (red shaded area).

(B) Nearest-neighbor plot in which lines are drawn from each tumor cell (red) to the nearest T cell (blue) and associated *G(r)* plot from a tumor with a low tumor-to-T cell spatial proximity score (Tm_T.SPS).

(C) Nearest-neighbor plot in which lines are drawn from each tumor cell (red) to the nearest T cell (blue) and associated *G(r)* plot from a tumor with a high tumor-to-T cell spatial proximity score (Tm_T.SPS).

(D) Boxplot illustrating significantly higher Tm_T.SPS in patients who achieved a pCR. The data are depicted as individual dots for each sample, along with the median, first, and third quartile (*n* = 54 patients). Statistical analysis was performed using the likelihood-ratio test. $^{**}p < 0.05$ (BH corrected).

(legend continued on next page)

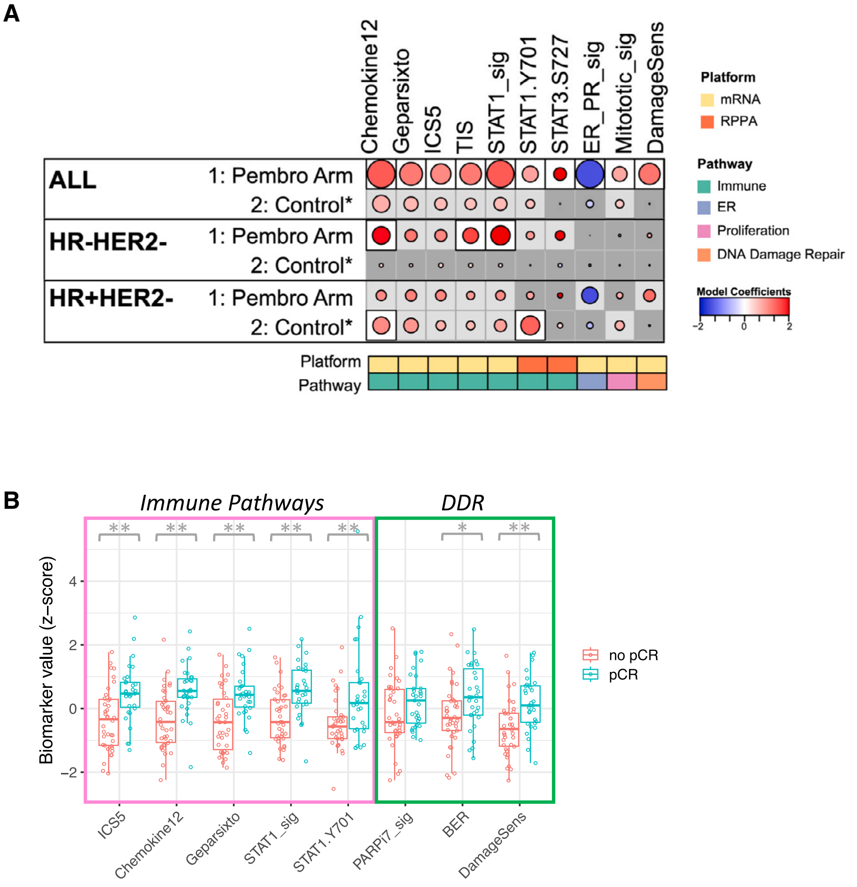

Figure 6. Immune signaling pathways and DNA damage and repair biomarkers associated with response to ICB

(A) Association dot matrix showing the level and direction of association between gene expression and RPPA biomarkers (columns) and pCR in the population/model as labeled (rows). Only those biomarkers that were significant ($p < 0.05$; after Benjamini-Hochberg multiple testing correction) in at least one cohort are shown. See Figure 3 legend for number of patients in each cohort (mRNA and RPPA platforms). Color of dot indicates direction of association (red, higher in pCR; blue, higher in non-pCR). Size of dot is proportional to significance (larger dots → smaller p values). Background square color indicates: BH FDR $p < 0.05$ (white), nominal $p < 0.05$ (light gray), not significant (dark gray).

(B) Boxplots illustrating the associations of various biomarkers related to immune pathways and DNA damage/repair with pCR in the Pembro arm. The data are depicted as individual dots for each sample, along with the median, first, and third quartile. Statistical analysis was performed using the likelihood-ratio test. *$p < 0.05$ (not corrected), **$p < 0.05$ (BH corrected). See also Figures S6 and S7 and Tables S2, S3, S4, S5, and S6.

immune microenvironments defined by the Immune RPS were significantly associated with response to pembrolizumab.

DISCUSSION

Immunotherapy with ICIs has shown promise as a therapeutic strategy for breast cancer. Characterization of the tumor immune microenvironment will facilitate our understanding of the mechanisms through which these cancers respond or fail to respond to immune checkpoint blockade. In addition to immune cell counts, evaluating spatial relationships between tumor and immune cells, as well as between different populations of immune cells, will be essential to this understanding. In this study, we examined associations between baseline immune markers and response to anti-PD-1 neoadjuvant therapy. We evaluated biomarkers in pretreatment biopsy samples on three platforms: gene expression arrays, RPPAs, and multiplex immunofluorescence on formalin-fixed and paraffin-embedded (FFPE) tissue sections. This provided a unique opportunity to investigate immunological correlates of response to ICI therapy.

Responsiveness to anti-PD-1 has been correlated with PD-L1 expression in some cancers, but PD-L1 IHC of tumor and/or immune cells has produced inconsistent results in others. Herein, we used multiplex immunofluorescence to quantitate cell densities along with spatial metrics for cell-to-cell proximity and found several measurements of the PD-1/PD-L1 axis that associated with response to therapy. Since PD-1 is the direct target of anti-PD-1 agents such as pembrolizumab, it seems reasonable that the expression of PD-1 in the tumor immune microenvironment would be associated with response to anti-PD-1 therapy. We found expression of the PD-1 gene (PDCD1) to be associated with response in the overall cohort and PD-1^{+} cells (by mIF)

(E) Nearest-neighbor plot in which lines are drawn from each PD-1^{+} T cell (green) to the nearest PD-L1^{+} cell (magenta) and associated *G(r)* plot from a tumor with a low PD-1^{+} T cell to PD-L1^{+} cell spatial proximity score (PD1T_PDL1.SPS).

(F) Nearest-neighbor plot in which lines are drawn from each PD-1^{+} T cell (green) to the nearest PD-L1^{+} cell (magenta) and associated *G(r)* plot from a tumor with a high PD-1^{+} T cell to PD-L1^{+} cell spatial proximity score (PD1T_PDL1.SPS).

(G) Boxplot illustrating significantly higher PD1T_PDL1.SPS in patients who achieved a pCR. The data are depicted as individual dots for each sample, along with the median, first, and third quartile ($n = 54$ patients). Statistical analysis was performed using the likelihood-ratio test. **$p < 0.05$ (BH corrected).

(H) Association dot matrix showing the level and direction of association between spatial proximity scores (columns) and pCR in the population/model as labeled (rows). Only those biomarkers that were significant ($p < 0.05$; after Benjamini-Hochberg multiple testing correction) in at least one cohort are shown. See Figure 3 legend for number of patients in each cohort (multi-IF platform). Color of dot indicates direction of association (red, higher in pCR; blue, higher in non-pCR). Size of dot is proportional to significance (larger dots → smaller p values). Background square color indicates: BH FDR $p < 0.05$ (white), nominal $p < 0.05$ (light gray), not significant (dark gray). See also Figure S5 and Tables S4 and S5.

A

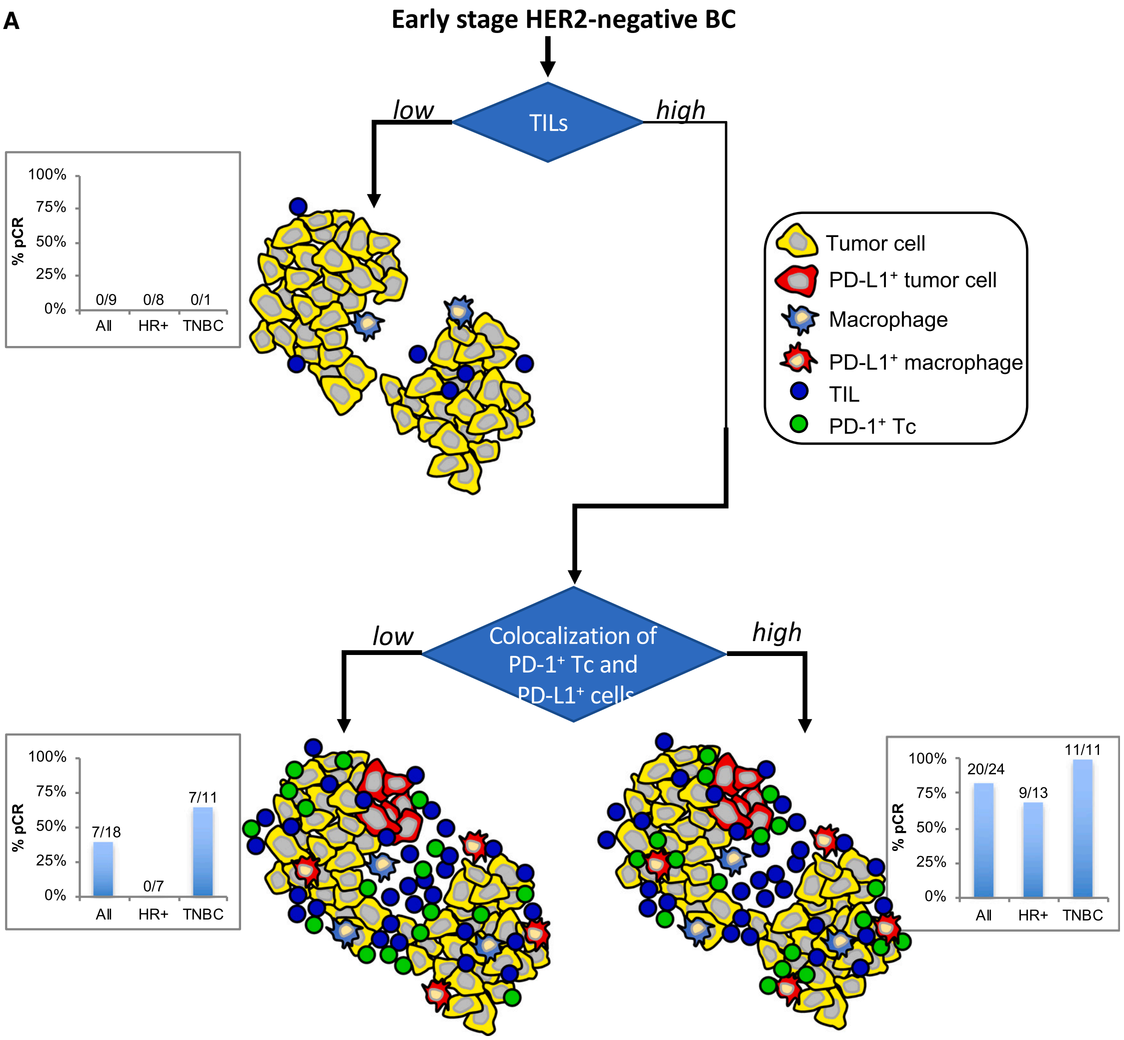

B

	TIL low	TIL high PD1Tc_PDL1 low	TIL high PD1Tc_PDL1 high
no pCR	9	11	4
pCR	0	7	20

$p < 0.00001$

C

	RPS Immune-	RPS Immune+
no pCR	19	5
pCR	4	23

$p < 0.00001$

Figure 7. Illustration of different tumor immune microenvironments as characterized by mIF and their associated pCR rates, overall and by receptor subtypes

(A) TILs were defined as $CD3^+$ T cells plus $CD20^+$ B cells. The cut-point for low/high TILs was set at 12.5% (percent of total cell counts). Colocalization of PD-1^+Tc and PD-$L1^+$ cells (immune or tumor) was defined using the Morisita-Horn index. A median cut-point was used to define low/high colocalization scores. Bar graphs indicate pCR rates for the Pembro arm, all cases or split out by receptor subtypes, for each of the depicted immune microenvironments: low TILs, high TILs, high TILs & low colocalization score, high TILs & high colocalization score.

(B) Immune microenvironments defined by percent TILs and colocalization of PD-1^+Tc with PD-$L1^+$ cells (immune or tumor) are significantly associated with response (Fisher exact test).

(C) Immune microenvironments defined by immune response predictive subtype (RPS) are significantly associated with response (Fisher exact test).

associated with response in HR^+HER2^- patients. On the PD-L1 side of the axis, we found expression of the PD-L1 gene (CD274), PD-$L1^+$ immune cells (in particular macrophages), and the PD-L1 CPS, but not PD-$L1^+$ tumor cells or the PD-L1 TPS associated with pCR in the overall cohort. These findings are consistent with other reports of PD-L1 expression on immune cells, but not tumor cells, being associated with response to anti-PD-1 therapy.[23,24] In addition, PD-$L1^+$ macrophages have been shown to play a key role in response to immune checkpoint blockade in mouse models[25,26] and predict immunotherapy outcomes in

patients with NSCLC and melanoma.[27,28] Further studies exploring PD-L1 expression on lymphocyte subsets and PD-1/PD-L1 double-positive lymphocytes may add to these findings.

Anti-PD-1 agents work by disrupting the interaction of PD-1 and PD-L1. Thus, a biomarker of this interaction, rather than the individual PD-1^+ and PD-$L1^+$ cell densities, may provide a useful marker of response. Perhaps one of the most relevant findings in this study is the observation that colocalization or close spatial proximity of PD-1^+ T cells (and/or PD-1^+CD8^+ Tc) with PD-$L1^+$ cells (immune and/or tumor cells) was significantly associated with response in the overall cohort as well as the TN and HR^+HER2^- subtypes. Similar PD-1/PD-L1 proximity results have been reported in melanoma[29,30] and Merkel cell carcinoma[31] in which measurements of PD-1^+ cells in close proximity to PD-$L1^+$ cells were associated with clinical response to anti-PD-1 therapy. Additionally, close proximity of CD8^+ T cells with PD-$L1^+$ cells, as well as any PD1^+ cell with PD-$L1^+$ cells, was prognostic for poor overall survival in oropharyngeal squamous cell carcinoma.[32]

Previous studies have suggested that CD4^+ T cells may play a role equivalent to or possibly greater than CD8^+ T cells in mediating the efficacy of immunotherapy.[33–35] Although we did not stain directly for the presence of CD4^+ T cells, we did find that CD8^- T cells, as well as a Th1 GES, were associated with pCR. Recent clinical evidence supports the importance of CD4^+ T cells in generating successful anti-tumor immunity. Single-cell RNA sequencing and T cell receptor analyses from colorectal cancer biopsies demonstrated that microsatellite-instable tumors (which are more likely to respond to ICIs) showed a preferential enrichment of a Th1-like subset of CD4^+ T cells.[36] In addition, two recent studies have demonstrated that the presence of specific subsets of CD4^+ T cells in the peripheral blood of NSCLC patients treated with PD-L1/PD-1 blockade therapy was significantly correlated with better responses.[37,38] In other studies, CD4 count measured using digital spatial profiling was an immune parameter associated with positive outcomes in melanoma[39] and NSCLC[23] patients treated with ICIs.

The identification of a DC GES associated with pCR in our study is in line with recent findings regarding the role of DCs in immunotherapy with ICIs. In mouse models, Garris and colleagues showed that effective anti-PD-1 immunotherapy required a subset of tumor-infiltrating DCs.[40] In human melanomas, the presence of DC has been shown to correlate with response to anti-PD-1 therapy.[41] Patients with renal cell carcinoma with a high DC GES at baseline were more likely to respond to atezolizumab (anti-PD-L1) therapy.[42] Similar observations were made in atezolizumab-treated patients with NSCLC.[42] In contrast, there was no significant difference in response between high- and low-DC-signature groups in patients treated with chemotherapy only, similar to our results. Finally, DCs in human breast cancers have been shown to express CXCL9,[43] and CXCL9 expression correlates with response to anti-PD-1 therapy,[44] further suggesting a role for DCs in the context of patient response to anti-PD-1.

The identification of a mast cell GES as a predictor of resistance to anti-PD-1 therapy in HR^+HER2^- patients is also an interesting finding. This is consistent with a recent report demonstrating that tumor-infiltrating mast cells are associated with resistance to anti-PD-1 therapy in a humanized mouse melanoma model.[45] Combining anti-PD-1 with sunitinib or imatinib resulted in the depletion of mast cells and complete regression of tumors in this model. Additionally, recent clinical studies found that having a lower pretreatment mast cell density was significantly associated with achieving a pCR to neoadjuvant chemotherapy in breast cancer[46]. Similarly, we observed a negative association between an MC GES and pCR in the control chemotherapy arm, although this was not significant.

Five immune signaling GESs were associated with response to Pembro (Chemokine12, GeparSixto, ICS5, TIS, and STAT1_sig). These signatures were highly correlated with each other suggesting they measure a similar state of immune activation in the tumor microenvironment. The Chemokine12 gene signature, which consists of a panel of 12 chemokine genes, was identified to strongly predict the presence of ectopic lymph node-like structures (TLSs) in primary colorectal carcinomas.[47] This gene signature has also been evaluated in invasive breast cancer where it was also associated with the presence of TLS and better survival outcomes.[48] TLSs have the appearance of typical peripheral lymph nodes and as such display a focal concentration of T cells and B cells. Although we did not measure TLS directly, we did observe positive correlations between the Chemokine12 gene signature and T cell/B cell spatial metrics (MH colocalization indices and T_B SPSs).

Finally, despite FDA approval of pembrolizumab for treatment of microsatellite instability-high and mismatch repair-deficient cancers, interestingly we found that a number of DNA repair protein/phosphoprotein biomarkers, including phosphorylated BRCA1, CHK1, and CHK2, along with cleaved PARP; apoptosis/DNA damage markers cleaved caspase-3 and caspase-9 and phosphorylated gH2AX; and microsatellite instability markers MLH1, MSH6, and MSH2 did not associate with response to pembrolizumab in either TNBC or HR+/$HER2^-$ tumors in this study.

Overall, of the 126 biomarkers evaluated across three platforms, 56 were significantly associated with response to pembrolizumab (13 expression signatures, 6 RPPA markers, 37 mIF markers). Many of these biomarkers were correlated with each other (Figure S2). For example, the immune signaling GESs, the immune cell densities identified by mIF, and the immune cell population GESs were all correlated with each other with only a few exceptions. In contrast, the PD-1/PD-L1-related spatial metrics, while correlated with each other, did not generally correlate with the immune signaling signatures or immune cell populations (mIF or expression signatures). These results suggest that the PD-1/PD-L1 spatial metrics are measuring a different aspect of the tumor immune microenvironment.

In summary, we identified several mIF markers and gene expression markers, representing immune cell populations as well as tumor epithelium-based proteomic/phosphoproteomic RPPA-generated biomarkers that were significantly associated with response in the pembrolizumab arm in the population as a whole. Interestingly, when examined by receptor subtypes, most of these associations were significant in the HR^+HER2^- cohort (including T cells, B cells, and PD1^+ cells, and as previously reported tumor epithelium expressed MHC II[49] but not

the TN subgroup. In contrast, GESs related to chemokines (Chemokine12), inflammation (TIS), or immune signaling (STAT1) predicted response to pembrolizumab in TN but not $HR^{+}HER2^{-}$ tumors. These results indicate that within the receptor subtypes there are differences in the immune microenvironments related to response to immune checkpoint blockade. Indeed, we have recently shown that both $HR^{+}HER2^{-}$ and TN tumors can be split into $Immune^{+}$ and $Immune^{-}$ RPSs based on GESs where the $Immune^{+}$ tumors have a higher likelihood of responding to immunotherapy.[22] This study illustrates the potential of spatial-based multiplex immunofluorescence staining of tumor tissue, as well as GESs and phosphoprotein/protein pathway analysis, as tools to discover biomarkers of response to immunotherapy in the neoadjuvant setting.

Limitations of the study

This study has to be interpreted in the context of its limitations. First, although the adaptive design of the I-SPY 2 trial allows for efficient and rapid identification of promising agent/subtype combinations based on their estimated likelihood of phase 3 success, this has the unfortunate consequence, from a biomarker perspective, of producing unbalanced groups with low patient numbers in each arm. As none of the concurrent controls had FFPE tissue samples available (only frozen tissue), which were required for the mIF assays, FFPE samples from a cohort of non-concurrent controls from the latter part of the trial were utilized for mIF assays. Although these samples were matched based on receptor subtypes, MammaPrint scores, and response to therapy, this limited some comparisons. Finally, we assessed multiple hypotheses in this study, and, although we applied statistical correction for multiple testing, this does not preclude the need for validation in independent cohorts. As such, the results presented here must be considered exploratory/hypothesis generating.

RESOURCE AVAILABILITY

Lead contact

Further information and requests for resources or data should be directed to Michael Campbell (Michael.Campbell@ucsf.edu).

Materials availability

Requests for sharing of materials should be directed to the lead contact.

Data and code availability

- Transcriptomic, protein/phosphoprotein, and clinical data used in this study are available in NCBI's Gene Expression Omnibus (GEO) SuperSeries GSE196096 (https://www.ncbi.nlm.nih.gov/geo/query/acc.cgi?acc=GSE196096) and its two SubSeries GSE194040 (mRNA: https://www.ncbi.nlm.nih.gov/geo/query/acc.cgi?acc=GSE194040) and GSE196093 (RPPA: https://www.ncbi.nlm.nih.gov/geo/query/acc.cgi?acc=GSE196093), and through the I-SPY2 Google Cloud repository (www.ispytrials.org/results/data) as part of the I-SPY2-990 mRNA/RPPA data resource. Patient-level scores for the 126 biomarkers presented herein including mIF features, GESs, and RPPA endpoints are available in Tables S5 (mIF) and S6 (mRNA and RPPA), along with clinical variables HR (1 = HR+; 0 = HR-), HER2 (1 = HER2+; 0 = $HER2^{-}$), Tx arm, and pCR (1 = yes; 0 = no). Additional de-identified subject level data may be requested by qualified investigators. Details of the trial, data, contact information, proposal forms, and review and approval process are available at the following website: https://www.I-SPYtrials.org/collaborate/proposal-submissions.
- Software packages used in this study are listed in the key resources table. R scripts used in this work have been deposited at Zenodo (https://doi.org/10.5281/zenodo.13323763).
- Any additional information required to reanalyze the data reported in this paper is available from the lead contact upon request.

ACKNOWLEDGMENTS

This study was conducted with support from Quantum Leap Healthcare Collaborative, NIH/NCI, Safeway (an Albertsons Company), and the Breast Cancer Research Foundation. We offer sincere thanks to our Biomarker Working Group, our investigators, our wonderful advocates, and all the patients who volunteered to participate in the I-SPY2 trial.

AUTHOR CONTRIBUTIONS

M.J.C., D.M.W., C.Y., L.v.V., J.W., E.F.P., and L.E. designed the study, interpreted the data, and prepared the manuscript. M.J.C., H.M., A.B., and C.H. designed multiplex panels. M.J.C., J.B., S.V., and C.H. carried out mIF tissue staining and image analyses. D.M.W., C.Y., and M.J.C. analyzed the data with assistance from Z.Z. L.B.-S. leads the I-SPY lab, overseeing tissue sectioning and molecular assays. J.W., I.R.G., and E.F.P. generated the RPPA data. G.L.H. is the I-SPY 2 trial Biomarker Liaison and manages the Biomarker Working Group, led by L.v.V., with members M.J.C., D.M.W., C.Y., L.B.-S., J.W., E.F.P., and M.C.L. L.S. manages the I-SPY2/22 P01. J.P. is a patient advocate. S.M.A. managed the I-SPY trials operations. A.M.D., R.N., L.P., N.M.H., and D.Y. are I-SPY2 working group leads. L.E., D.A.B., and N.M.H. are the principal investigators of I-SPY2. I-SPY2 trial investigators and biomarker and other working group members participated in all aspects of the trial and contributed to its success. All of the authors participated in manuscript review.

DECLARATION OF INTERESTS

J.W. reports honoraria from DAVA Oncology; consults for Baylor College of Medicine; has ownership in Theralink; and is co-inventor of the RPPA technology, and phospho-HER2 and -EGFR response predictors with filed patents. C.H. is an employee of Akoya Biosciences. R.N. reports grants from Quantum Leap, AstraZeneca, Celgene, Corcept Therapeutics, Genentech/Roche, Immunomedics, Merck, OBI Pharma, Odonate Therapeutics, Pfizer, and Seattle Genetics outside the submitted work; and personal fees from Aduro, AstraZeneca, Athenex, Celgene, Daiichi Sankyo, G1 Therapeutics, Genentech, MacroGenics, Merck, Novartis, Pfizer, Puma, and Syndax. M.C.L. reports support from Eisai, Genentech, GRAIL, Menarini Silicon Biosystems, Merck, Novartis, Seattle Genetics, and Tesaro. D.Y. reports unrelated support from Boehringer Ingelheim. A.M.D. reports honoraria or consulting for Pfizer and Context Therapeutics and reports support from Novartis, Pfizer, Genentech, Calithera, and Menarini. L.P. reports consulting fees and honoraria for advisory board participation from Pfizer, AstraZeneca, Merck, Novartis, Bristol-Myers Squibb, GlaxoSmithKline, Genentech/Roche, Personalis, Daiichi, Natera, and Exact Sciences and institutional research funding from Seagen, GlaxoSmithKline, AstraZeneca, Merck, Pfizer, and Bristol-Myers Squibb. D.A.B. is co-owner of Berry Consultants LLC, a company that designs adaptive clinical trials (including I-SPY2). E.F.P. reports leadership, stock/ownership, consulting/advisory, and travel funds from Perthera and Ceres Nanosciences; stock and consulting/advisory for Theralink Technologies, Inc; support from Ceres Nanosciences, GlaxoSmithKline, AbbVie, Symphogen, Genentech, SpringWorks Therapeutics, and Deciphera Therapeutics; and patents/royalties from NIH and filed patents for p-HER2 and p-EGFR response predictors. L.v.V. is a co-inventor of the MammaPrint signature and a part-time employee and stockholder of Agendia NV. L.E. is an unpaid member of the board of directors of Quantum Leap Healthcare Collaborative (QLHC), has received grant support from QLHC for the I-SPY2 trial, and is on the Blue Cross/Blue Shield Medical Advisory Panel.

STAR★METHODS

Detailed methods are provided in the online version of this paper and include the following:

- KEY RESOURCES TABLE
- EXPERIMENTAL MODEL AND SUBJECT DETAILS
  - I-SPY 2 trial overview
  - Trial design
  - Eligibility
  - Treatment
  - Trial oversight
- METHOD DETAILS
  - Pretreatment biopsy processing
  - Multiplex immunofluorescence tumor tissue profiling
  - mIF image analysis
  - mIF spatial distribution analysis
  - Gene expression profiling
  - Protein/phospho-protein profiling
  - Biomarkers evaluated
- QUANTIFICATION AND STATISTICAL ANALYSIS
  - pCR association biomarker analysis

SUPPLEMENTAL INFORMATION

Supplemental information can be found online at https://doi.org/10.1016/j.xcrm.2024.101799.

Received: July 25, 2022
Revised: May 13, 2024
Accepted: September 29, 2024
Published: November 6, 2024

REFERENCES

1. Burtness, B., Harrington, K.J., Greil, R., Soulières, D., Tahara, M., de Castro, G., Psyrri, A., Basté, N., Neupane, P., Bratland, Å., et al. (2019). Pembrolizumab alone or with chemotherapy versus cetuximab with chemotherapy for recurrent or metastatic squamous cell carcinoma of the head and neck (KEYNOTE-048): a randomised, open-label, phase 3 study. Lancet *394*, 1915–1928. https://doi.org/10.1016/S0140-6736(19)32591-7.
2. Larkin, J., Chiarion-Sileni, V., Gonzalez, R., Grob, J.-J., Rutkowski, P., Lao, C.D., Cowey, C.L., Schadendorf, D., Wagstaff, J., Dummer, R., et al. (2019). Five-Year Survival with Combined Nivolumab and Ipilimumab in Advanced Melanoma. N. Engl. J. Med. *381*, 1535–1546. https://doi.org/10.1056/NEJMoa1910836.
3. Motzer, R.J., Tannir, N.M., McDermott, D.F., Arén Frontera, O., Melichar, B., Choueiri, T.K., Plimack, E.R., Barthélémy, P., Porta, C., George, S., et al. (2018). Nivolumab plus Ipilimumab versus Sunitinib in Advanced Renal-Cell Carcinoma. N. Engl. J. Med. *378*, 1277–1290. https://doi.org/10.1056/NEJMoa1712126.
4. Reck, M., Rodríguez-Abreu, D., Robinson, A.G., Hui, R., Csőszi, T., Fülöp, A., Gottfried, M., Peled, N., Tafreshi, A., Cuffe, S., et al. (2016). Pembrolizumab versus Chemotherapy for PD-L1-Positive Non-Small-Cell Lung Cancer. N. Engl. J. Med. *375*, 1823–1833. https://doi.org/10.1056/NEJMoa1606774.
5. Schmid, P., Cortes, J., Pusztai, L., McArthur, H., Kümmel, S., Bergh, J., Denkert, C., Park, Y.H., Hui, R., Harbeck, N., et al. (2020). Pembrolizumab for Early Triple-Negative Breast Cancer. N. Engl. J. Med. *382*, 810–821. https://doi.org/10.1056/NEJMoa1910549.
6. Loibl, S., Untch, M., Burchardi, N., Huober, J., Sinn, B.V., Blohmer, J.-U., Grischke, E.-M., Furlanetto, J., Tesch, H., Hanusch, C., et al. (2019). A randomised phase II study investigating durvalumab in addition to an anthracycline taxane-based neoadjuvant therapy in early triple-negative breast cancer: clinical results and biomarker analysis of GeparNuevo study. Ann. Oncol. *30*, 1279–1288. https://doi.org/10.1093/annonc/mdz158.
7. Gianni, L., Huang, C.-S., Egle, D., Bermejo, B., Zamagni, C., Thill, M., Anton, A., Zambelli, S., Bianchini, G., Russo, S., et al. (2020). Abstract GS3-04: Pathologic complete response (pCR) to neoadjuvant treatment with or without atezolizumab in triple negative, early high-risk and locally advanced breast cancer. NeoTRIPaPDL1 Michelangelo randomized study. Cancer Res. *80*, GS3-04. https://doi.org/10.1158/1538-7445.SABCS19-GS3-04.
8. Mittendorf, E.A., Zhang, H., Barrios, C.H., Saji, S., Jung, K.H., Hegg, R., Koehler, A., Sohn, J., Iwata, H., Telli, M.L., et al. (2020). Neoadjuvant atezolizumab in combination with sequential nab-paclitaxel and anthracycline-based chemotherapy versus placebo and chemotherapy in patients with early-stage triple-negative breast cancer (IMpassion031): a randomised, double-blind, phase 3 trial. Lancet *396*, 1090–1100. https://doi.org/10.1016/S0140-6736(20)31953-X.
9. Nanda, R., Liu, M.C., Yau, C., Shatsky, R., Pusztai, L., Wallace, A., Chien, A.J., Forero-Torres, A., Ellis, E., Han, H., et al. (2020). Effect of Pembrolizumab Plus Neoadjuvant Chemotherapy on Pathologic Complete Response in Women With Early-Stage Breast Cancer: An Analysis of the Ongoing Phase 2 Adaptively Randomized I-SPY2 Trial. JAMA Oncol. *6*, 676–684. https://doi.org/10.1001/jamaoncol.2019.6650.
10. Binnewies, M., Roberts, E.W., Kersten, K., Chan, V., Fearon, D.F., Merad, M., Coussens, L.M., Gabrilovich, D.I., Ostrand-Rosenberg, S., Hedrick, C.C., et al. (2018). Understanding the tumor immune microenvironment (TIME) for effective therapy. Nat. Med. *24*, 541–550. https://doi.org/10.1038/s41591-018-0014-x.
11. Ayers, M., Lunceford, J., Nebozhyn, M., Murphy, E., Loboda, A., Kaufman, D.R., Albright, A., Cheng, J.D., Kang, S.P., Shankaran, V., et al. (2017). IFN-γ-related mRNA profile predicts clinical response to PD-1 blockade. J. Clin. Invest. *127*, 2930–2940. https://doi.org/10.1172/JCI91190.
12. Sinn, B.V., Loibl, S., Hanusch, C.A., Zahm, D.-M., Sinn, H.-P., Untch, M., Weber, K., Karn, T., Becker, C., Marmé, F., et al. (2021). Immune-related Gene Expression Predicts Response to Neoadjuvant Chemotherapy but not Additional Benefit from PD-L1 Inhibition in Women with Early Triple-negative Breast Cancer. Clin. Cancer Res. *27*, 2584–2591. https://doi.org/10.1158/1078-0432.CCR-20-3113.
13. Goodman, A.M., Kato, S., Bazhenova, L., Patel, S.P., Frampton, G.M., Miller, V., Stephens, P.J., Daniels, G.A., and Kurzrock, R. (2017). Tumor Mutational Burden as an Independent Predictor of Response to Immunotherapy in Diverse Cancers. Mol. Cancer Therapeut. *16*, 2598–2608. https://doi.org/10.1158/1535-7163.MCT-17-0386.
14. Davis, A.A., and Patel, V.G. (2019). The role of PD-L1 expression as a predictive biomarker: an analysis of all US Food and Drug Administration (FDA) approvals of immune checkpoint inhibitors. J. Immunother. Cancer *7*, 278. https://doi.org/10.1186/s40425-019-0768-9.
15. Carbone, D.P., Reck, M., Paz-Ares, L., Creelan, B., Horn, L., Steins, M., Felip, E., van den Heuvel, M.M., Ciuleanu, T.-E., Badin, F., et al. (2017). First-Line Nivolumab in Stage IV or Recurrent Non-Small-Cell Lung Cancer. N. Engl. J. Med. *376*, 2415–2426. https://doi.org/10.1056/NEJMoa1613493.
16. Mok, T.S.K., Wu, Y.-L., Kudaba, I., Kowalski, D.M., Cho, B.C., Turna, H.Z., Castro, G., Srimuninnimit, V., Laktionov, K.K., Bondarenko, I., et al. (2019). Pembrolizumab versus chemotherapy for previously untreated, PD-L1-expressing, locally advanced or metastatic non-small-cell lung cancer (KEYNOTE-042): a randomised, open-label, controlled, phase 3 trial. Lancet *393*, 1819–1830. https://doi.org/10.1016/S0140-6736(18)32409-7.
17. Rimm, D.L., Han, G., Taube, J.M., Yi, E.S., Bridge, J.A., Flieder, D.B., Homer, R., West, W.W., Wu, H., Roden, A.C., et al. (2017). A Prospective, Multi-institutional, Pathologist-Based Assessment of 4 Immunohistochemistry Assays for PD-L1 Expression in Non-Small Cell Lung Cancer. JAMA Oncol. *3*, 1051–1058. https://doi.org/10.1001/jamaoncol.2017.0013.
18. Reisenbichler, E.S., Han, G., Bellizzi, A., Bossuyt, V., Brock, J., Cole, K., Fadare, O., Hameed, O., Hanley, K., Harrison, B.T., et al. (2020).

Prospective multi-institutional evaluation of pathologist assessment of PD-L1 assays for patient selection in triple negative breast cancer. Mod. Pathol. *33*, 1746–1752. https://doi.org/10.1038/s41379-020-0544-x.

19. Rugo, H.S., Loi, S., Adams, S., Schmid, P., Schneeweiss, A., Barrios, C.H., Iwata, H., Diéras, V., Winer, E.P., Kockx, M.M., et al. (2021). PD-L1 Immunohistochemistry Assay Comparison in Atezolizumab plus nab-Paclitaxel-Treated Advanced Triple-Negative Breast Cancer. J. Natl. Cancer Inst. *113*, 1733–1743. https://doi.org/10.1093/jnci/djab108.
20. Stack, E.C., Wang, C., Roman, K.A., and Hoyt, C.C. (2014). Multiplexed immunohistochemistry, imaging, and quantitation: a review, with an assessment of Tyramide signal amplification, multispectral imaging and multiplex analysis. Methods *70*, 46–58. https://doi.org/10.1016/j.ymeth.2014.08.016.
21. Lu, S., Stein, J.E., Rimm, D.L., Wang, D.W., Bell, J.M., Johnson, D.B., Sosman, J.A., Schalper, K.A., Anders, R.A., Wang, H., et al. (2019). Comparison of Biomarker Modalities for Predicting Response to PD-1/PD-L1 Checkpoint Blockade: A Systematic Review and Meta-analysis. JAMA Oncol. *5*, 1195–1204. https://doi.org/10.1001/jamaoncol.2019.1549.
22. Wolf, D.M., Yau, C., Wulfkuhle, J., Brown-Swigart, L., Gallagher, R.I., Lee, P.R.E., Zhu, Z., Magbanua, M.J., Sayaman, R., O'Grady, N., et al. (2022). Redefining breast cancer subtypes to guide treatment prioritization and maximize response: Predictive biomarkers across 10 cancer therapies. Cancer Cell *40*, 609–623.e6. https://doi.org/10.1016/j.ccell.2022.05.005.
23. Zugazagoitia, J., Gupta, S., Liu, Y., Fuhrman, K., Gettinger, S., Herbst, R.S., Schalper, K.A., and Rimm, D.L. (2020). Biomarkers Associated with Beneficial PD-1 Checkpoint Blockade in Non-Small Cell Lung Cancer (NSCLC) Identified Using High-Plex Digital Spatial Profiling. Clin. Cancer Res. *26*, 4360–4368. https://doi.org/10.1158/1078-0432.CCR-20-0175.
24. Ahmed, F.S., Gaule, P., McGuire, J., Patel, K., Blenman, K., Pusztai, L., and Rimm, D.L. (2020). PD-L1 Protein Expression on Both Tumor Cells and Macrophages are Associated with Response to Neoadjuvant Durvalumab with Chemotherapy in Triple-negative Breast Cancer. Clin. Cancer Res. *26*, 5456–5461. https://doi.org/10.1158/1078-0432.CCR-20-1303.
25. Lin, H., Wei, S., Hurt, E.M., Green, M.D., Zhao, L., Vatan, L., Szeliga, W., Herbst, R., Harms, P.W., Fecher, L.A., et al. (2018). Host expression of PD-L1 determines efficacy of PD-L1 pathway blockade-mediated tumor regression. J. Clin. Invest. *128*, 805–815. https://doi.org/10.1172/JCI96113.
26. Tang, H., Liang, Y., Anders, R.A., Taube, J.M., Qiu, X., Mulgaonkar, A., Liu, X., Harrington, S.M., Guo, J., Xin, Y., et al. (2018). PD-L1 on host cells is essential for PD-L1 blockade-mediated tumor regression. J. Clin. Invest. *128*, 580–588. https://doi.org/10.1172/JCI96061.
27. Liu, Y., Zugazagoitia, J., Ahmed, F.S., Henick, B.S., Gettinger, S.N., Herbst, R.S., Schalper, K.A., and Rimm, D.L. (2020). Immune Cell PD-L1 Colocalizes with Macrophages and Is Associated with Outcome in PD-1 Pathway Blockade Therapy. Clin. Cancer Res. *26*, 970–977. https://doi.org/10.1158/1078-0432.CCR-19-1040.
28. Toki, M.I., Merritt, C.R., Wong, P.F., Smithy, J.W., Kluger, H.M., Syrigos, K.N., Ong, G.T., Warren, S.E., Beechem, J.M., and Rimm, D.L. (2019). High-Plex Predictive Marker Discovery for Melanoma Immunotherapy-Treated Patients Using Digital Spatial Profiling. Clin. Cancer Res. *25*, 5503–5512. https://doi.org/10.1158/1078-0432.CCR-19-0104.
29. Johnson, D.B., Bordeaux, J., Kim, J.Y., Vaupel, C., Rimm, D.L., Ho, T.H., Joseph, R.W., Daud, A.I., Conry, R.M., Gaughan, E.M., et al. (2018). Quantitative Spatial Profiling of PD-1/PD-L1 Interaction and HLA-DR/IDO-1 Predicts Improved Outcomes of Anti-PD-1 Therapies in Metastatic Melanoma. Clin. Cancer Res. *24*, 5250–5260. https://doi.org/10.1158/1078-0432.CCR-18-0309.
30. Tumeh, P.C., Harview, C.L., Yearley, J.H., Shintaku, I.P., Taylor, E.J.M., Robert, L., Chmielowski, B., Spasic, M., Henry, G., Ciobanu, V., et al. (2014). PD-1 blockade induces responses by inhibiting adaptive immune resistance. Nature *515*, 568–571. https://doi.org/10.1038/nature13954.
31. Giraldo, N.A., Nguyen, P., Engle, E.L., Kaunitz, G.J., Cottrell, T.R., Berry, S., Green, B., Soni, A., Cuda, J.D., Stein, J.E., et al. (2018). Multidimensional, quantitative assessment of PD-1/PD-L1 expression in patients with Merkel cell carcinoma and association with response to pembrolizumab. J. Immunother. Cancer *6*, 99. https://doi.org/10.1186/s40425-018-0404-0.
32. Tsakiroglou, A.M., Fergie, M., Oguejiofor, K., Linton, K., Thomson, D., Stern, P.L., Astley, S., Byers, R., and West, C.M.L. (2020). Spatial proximity between T and PD-L1 expressing cells as a prognostic biomarker for oropharyngeal squamous cell carcinoma. Br. J. Cancer *122*, 539–544. https://doi.org/10.1038/s41416-019-0634-z.
33. Binnewies, M., Mujal, A.M., Pollack, J.L., Combes, A.J., Hardison, E.A., Barry, K.C., Tsui, J., Ruhland, M.K., Kersten, K., Abushawish, M.A., et al. (2019). Unleashing Type-2 Dendritic Cells to Drive Protective Antitumor CD4^{+} T Cell Immunity. Cell *177*, 556–571.e16. https://doi.org/10.1016/j.cell.2019.02.005.
34. Spitzer, M.H., Carmi, Y., Reticker-Flynn, N.E., Kwek, S.S., Madhireddy, D., Martins, M.M., Gherardini, P.F., Prestwood, T.R., Chabon, J., Bendall, S.C., et al. (2017). Systemic Immunity Is Required for Effective Cancer Immunotherapy. Cell *168*, 487–502.e15. https://doi.org/10.1016/j.cell.2016.12.022.
35. Tay, R.E., Richardson, E.K., and Toh, H.C. (2021). Revisiting the role of CD4^{+} T cells in cancer immunotherapy-new insights into old paradigms. Cancer Gene Ther. *28*, 5–17. https://doi.org/10.1038/s41417-020-0183-x.
36. Zhang, L., Yu, X., Zheng, L., Zhang, Y., Li, Y., Fang, Q., Gao, R., Kang, B., Zhang, Q., Huang, J.Y., et al. (2018). Lineage tracking reveals dynamic relationships of T cells in colorectal cancer. Nature *564*, 268–272. https://doi.org/10.1038/s41586-018-0694-x.
37. Kagamu, H., Kitano, S., Yamaguchi, O., Yoshimura, K., Horimoto, K., Kitazawa, M., Fukui, K., Shiono, A., Mouri, A., Nishihara, F., et al. (2020). CD4^{+} T-cell Immunity in the Peripheral Blood Correlates with Response to Anti-PD-1 Therapy. Cancer Immunol. Res. *8*, 334–344. https://doi.org/10.1158/2326-6066.CIR-19-0574.
38. Zuazo, M., Arasanz, H., Fernández-Hinojal, G., García-Granda, M.J., Gato, M., Bocanegra, A., Martínez, M., Hernández, B., Teijeira, L., Morilla, I., et al. (2019). Functional systemic CD4 immunity is required for clinical responses to PD-L1/PD-1 blockade therapy. EMBO Mol. Med. *11*, e10293. https://doi.org/10.15252/emmm.201910293.
39. Amaria, R.N., Reddy, S.M., Tawbi, H.A., Davies, M.A., Ross, M.I., Glitza, I.C., Cormier, J.N., Lewis, C., Hwu, W.-J., Hanna, E., et al. (2018). Neoadjuvant immune checkpoint blockade in high-risk resectable melanoma. Nat. Med. *24*, 1649–1654. https://doi.org/10.1038/s41591-018-0197-1.
40. Garris, C.S., Arlauckas, S.P., Kohler, R.H., Trefny, M.P., Garren, S., Piot, C., Engblom, C., Pfirschke, C., Siwicki, M., Gungabeesoon, J., et al. (2018). Successful Anti-PD-1 Cancer Immunotherapy Requires T Cell-Dendritic Cell Crosstalk Involving the Cytokines IFN-γ and IL-12. Immunity *49*, 1148–1161.e7. https://doi.org/10.1016/j.immuni.2018.09.024.
41. Barry, K.C., Hsu, J., Broz, M.L., Cueto, F.J., Binnewies, M., Combes, A.J., Nelson, A.E., Loo, K., Kumar, R., Rosenblum, M.D., et al. (2018). A natural killer-dendritic cell axis defines checkpoint therapy-responsive tumor microenvironments. Nat. Med. *24*, 1178–1191. https://doi.org/10.1038/s41591-018-0085-8.
42. Mayoux, M., Roller, A., Pulko, V., Sammicheli, S., Chen, S., Sum, E., Jost, C., Fransen, M.F., Buser, R.B., Kowanetz, M., et al. (2020). Dendritic cells dictate responses to PD-L1 blockade cancer immunotherapy. Sci. Transl. Med. *12*, eaav7431. https://doi.org/10.1126/scitranslmed.aav7431.
43. de Mingo Pulido, Á., Gardner, A., Hiebler, S., Soliman, H., Rugo, H.S., Krummel, M.F., Coussens, L.M., and Ruffell, B. (2018). TIM-3 Regulates CD103^{+} Dendritic Cell Function and Response to Chemotherapy in Breast Cancer. Cancer Cell *33*, 60–74.e6. https://doi.org/10.1016/j.ccell.2017.11.019.
44. Chow, M.T., Ozga, A.J., Servis, R.L., Frederick, D.T., Lo, J.A., Fisher, D.E., Freeman, G.J., Boland, G.M., and Luster, A.D. (2019). Intratumoral Activity of the CXCR3 Chemokine System Is Required for the Efficacy of Anti-PD-1 Therapy. Immunity *50*, 1498–1512.e5. https://doi.org/10.1016/j.immuni.2019.04.010.

45. Somasundaram, R., Connelly, T., Choi, R., Choi, H., Samarkina, A., Li, L., Gregorio, E., Chen, Y., Thakur, R., Abdel-Mohsen, M., et al. (2021). Tumor-infiltrating mast cells are associated with resistance to anti-PD-1 therapy. Nat. Commun. *12*, 346. https://doi.org/10.1038/s41467-020-20600-7.

46. Reddy, S.M., Reuben, A., Barua, S., Jiang, H., Zhang, S., Wang, L., Gopalakrishnan, V., Hudgens, C.W., Tetzlaff, M.T., Reuben, J.M., et al. (2019). Poor Response to Neoadjuvant Chemotherapy Correlates with Mast Cell Infiltration in Inflammatory Breast Cancer. Cancer Immunol. Res. *7*, 1025–1035. https://doi.org/10.1158/2326-6066.CIR-18-0619.

47. Coppola, D., Nebozhyn, M., Khalil, F., Dai, H., Yeatman, T., Loboda, A., and Mulé, J.J. (2011). Unique ectopic lymph node-like structures present in human primary colorectal carcinoma are identified by immune gene array profiling. Am. J. Pathol. *179*, 37–45. https://doi.org/10.1016/j.ajpath.2011.03.007.

48. Prabhakaran, S., Rizk, V.T., Ma, Z., Cheng, C.-H., Berglund, A.E., Coppola, D., Khalil, F., Mulé, J.J., and Soliman, H.H. (2017). Evaluation of invasive breast cancer samples using a 12-chemokine gene expression score: correlation with clinical outcomes. Breast Cancer Res. *19*, 71. https://doi.org/10.1186/s13058-017-0864-z.

49. Gonzalez-Ericsson, P.I., Wulfkhule, J.D., Gallagher, R.I., Sun, X., Axelrod, M.L., Sheng, Q., Luo, N., Gomez, H., Sanchez, V., Sanders, M., et al. (2021). Tumor-Specific Major Histocompatibility-II Expression Predicts Benefit to Anti-PD-1/L1 Therapy in Patients With HER2-Negative Primary Breast Cancer. Clin. Cancer Res. *27*, 5299–5306. https://doi.org/10.1158/1078-0432.CCR-21-0607.

50. Symmans, W.F., Wei, C., Gould, R., Yu, X., Zhang, Y., Liu, M., Walls, A., Bousamra, A., Ramineni, M., Sinn, B., et al. (2017). Long-Term Prognostic Risk After Neoadjuvant Chemotherapy Associated With Residual Cancer Burden and Breast Cancer Subtype. J. Clin. Oncol. *35*, 1049–1060. https://doi.org/10.1200/JCO.2015.63.1010.

51. Cardoso, F., van't Veer, L.J., Bogaerts, J., Slaets, L., Viale, G., Delaloge, S., Pierga, J.-Y., Brain, E., Causeret, S., DeLorenzi, M., et al. (2016). 70-Gene Signature as an Aid to Treatment Decisions in Early-Stage Breast Cancer. N. Engl. J. Med. *375*, 717–729. https://doi.org/10.1056/NEJMoa1602253.

52. Berry, D.A. (2011). Adaptive clinical trials in oncology. Nat. Rev. Clin. Oncol. *9*, 199–207. https://doi.org/10.1038/nrclinonc.2011.165.

53. Park, J.W., Liu, M.C., Yee, D., Yau, C., van 't Veer, L.J., Symmans, W.F., Paoloni, M., Perlmutter, J., Hylton, N.M., Hogarth, M., et al. (2016). Adaptive Randomization of Neratinib in Early Breast Cancer. N. Engl. J. Med. *375*, 11–22. https://doi.org/10.1056/NEJMoa1513750.

54. Rugo, H.S., Olopade, O.I., DeMichele, A., Yau, C., van 't Veer, L.J., Buxton, M.B., Hogarth, M., Hylton, N.M., Paoloni, M., Perlmutter, J., et al. (2016). Adaptive Randomization of Veliparib-Carboplatin Treatment in Breast Cancer. N. Engl. J. Med. *375*, 23–34. https://doi.org/10.1056/NEJMoa1513749.

55. Mori, H., Bolen, J., Schuetter, L., Massion, P., Hoyt, C.C., VandenBerg, S., Esserman, L., Borowsky, A.D., and Campbell, M.J. (2020). Characterizing the Tumor Immune Microenvironment with Tyramide-Based Multiplex Immunofluorescence. J. Mammary Gland Biol. Neoplasia *25*, 417–432. https://doi.org/10.1007/s10911-021-09479-2.

56. Francis, K., and Palsson, B.O. (1997). Effective intercellular communication distances are determined by the relative time constants for cyto/chemokine secretion and diffusion. Proc. Natl. Acad. Sci. USA *94*, 12258–12262. https://doi.org/10.1073/pnas.94.23.12258.

57. Horn, H.S. (1966). Measurement of "overlap" in comparative ecological studies. Am. Nat. *100*, 419–424.

58. Wolda, H. (1981). Similarity indices, sample size and diversity. Oecologia *50*, 296–302. https://doi.org/10.1007/BF00344966.

59. Barua, S., Fang, P., Sharma, A., Fujimoto, J., Wistuba, I., Rao, A.U.K., and Lin, S.H. (2018). Spatial interaction of tumor cells and regulatory T cells correlates with survival in non-small cell lung cancer. Lung Cancer *117*, 73–79. https://doi.org/10.1016/j.lungcan.2018.01.022.

60. Carstens, J.L., Correa de Sampaio, P., Yang, D., Barua, S., Wang, H., Rao, A., Allison, J.P., LeBleu, V.S., and Kalluri, R. (2017). Spatial computation of intratumoral T cells correlates with survival of patients with pancreatic cancer. Nat. Commun. *8*, 15095. https://doi.org/10.1038/ncomms15095.

61. Wulfkuhle, J.D., Yau, C., Wolf, D.M., Vis, D.J., Gallagher, R.I., Brown-Swigart, L., Hirst, G., Voest, E.E., DeMichele, A., Hylton, N., et al. (2018). Evaluation of the HER/PI3K/AKT Family Signaling Network as a Predictive Biomarker of Pathologic Complete Response for Patients With Breast Cancer Treated With Neratinib in the I-SPY 2 TRIAL. JCO Precis. Oncol. *2*, PO.18.00024. https://doi.org/10.1200/PO.18.00024.

62. Wolf, D.M., Yau, C., Sanil, A., Glas, A., Petricoin, E., Wulfkuhle, J., Severson, T.M., Linn, S., Brown-Swigart, L., Hirst, G., et al. (2017). DNA repair deficiency biomarkers and the 70-gene ultra-high risk signature as predictors of veliparib/carboplatin response in the I-SPY 2 breast cancer trial. NPJ Breast Cancer *3*, 31. https://doi.org/10.1038/s41523-017-0025-7.

STAR★METHODS

KEY RESOURCES TABLE

REAGENT or RESOURCE	SOURCE	IDENTIFIER
Antibodies		
Anti-FoxP3 antibody; clone SP97	Spring Biosciences	RRID: AB_10658507
Anti-pan-cytokeratin; clone AE1/AE3	Dako	RRID: AB_2631307
Anti-CD20; clone L26	Ventana/Roche	RRID: AB_2335956
Anti-CD3; clone 2GV6	Ventana/Roche	RRID: AB_2335978
Anti-Ki67; clone 30-9	Ventana/Roche	RRID: AB_2631262
Anti-CD117; clone D3W6Y	Cell Signaling	RRID: AB_2799120
Anti-PD-L1; clone E1L3N	Cell Signaling	RRID: AB_2687655
Anti-PD-1; clone EPR4877	Abcam	RRID: AB_2894867
Anti-CD8; clone 4B11	Leica	RRID: AB_442068
Anti-CD68; clone PG-M1	Dako	RRID: AB_2074844
Biological samples		
Tumor biopsy before treatment	I-SPY 2 trial	https://clinicaltrials.gov/ct2/show/NCT01042379
Critical commercial assays		
Custom Agilent 44K expression arrays	Agendia, Inc	https://www.ncbi.nlm.nih.gov/geo/query/acc.cgi?acc=GPL20078 https://www.ncbi.nlm.nih.gov/geo/query/acc.cgi?acc=GPL30483
MammaPrint	Agendia, Inc	https://agendia.com/mammaprint/
BluePrint	Agendia, Inc	https://agendia.com/blueprint/
Reverse phase protein array (RPPA)	Petricoin Lab, George Mason University	https://www.ncbi.nlm.nih.gov/geo/query/acc.cgi?acc=GPL28470
Deposited data		
Raw transcriptomic data	GEO accession number	*Gene Expression Omnibus (GEO)* SubSeries GSE194040 (mRNA), (https://www.ncbi.nlm.nih.gov/geo/query/acc.cgi?acc=GSE194040), within the SuperSeries GSE196096 (https://www.ncbi.nlm.nih.gov/geo/query/acc.cgi?acc=GSE196096)
Raw RPPA data	GEO accession number	*Gene Expression Omnibus (GEO)* SubSeries GSE196093 (RPPA) (https://www.ncbi.nlm.nih.gov/geo/query/acc.cgi?acc=GSE196093), within the SuperSeries GSE196096 (https://www.ncbi.nlm.nih.gov/geo/query/acc.cgi?acc=GSE196096).
Patient-level mIF features	This study	https://www.ispytrials.org/results/data
Patient-level expression signature scores	This study	https://www.ispytrials.org/results/data
Patient-level RPPA markers	This study	https://www.ispytrials.org/results/data
Software and algorithms		
stats R package (v.3.6.3)	R Core Team (2020)	https://stat.ethz.ch/R-manual/R-devel/library/stats/html/stats-package.html
lmtest R package (v.0.9–37)	Zeileis A, Hothorn T (2002). "Diagnostic Checking in Regression Relationships." R News, 2(3), 7–10.	https://CRAN.R-project.org/package=lmtest
rjags R package (v.4-10)	Martyn Plummer (2019). rjags: Bayesian Graphical Models using MCMC. R package v4-10.	https://CRAN.R-project.org/package=rjags

(*Continued on next page*)

Continued

REAGENT or RESOURCE	SOURCE	IDENTIFIER
phenoptr R package	Johnson K (2022). phenoptr: inForm Helper Functions. R package version 0.3.2	https://akoyabio.github.io/phenoptr/
spatstat R package	A. Baddeley, E. Rubak and R.Turner. Spatial Point Patterns: Methodology and Applications with R. Chapman and Hall/CRC Press, 2015.	https://spatstat.org/
divo R package	Christoph Sadee, Maciej Pietrzak, Michal Seweryn, Cankun Wang, Grzegorz Rempala (2019)	https://CRAN.R-project.org/package=divo
R scripts	N/A	Zenodo: https://doi.org/10.5281/zenodo.13323763

EXPERIMENTAL MODEL AND SUBJECT DETAILS

I-SPY 2 trial overview

I-SPY2 is an ongoing, open-label, adaptive, randomized phase II, multicenter trial of neoadjuvant therapy for early-stage breast cancer (NCT01042379; IND 105139). It is a platform trial evaluating multiple investigational arms in parallel against a common standard of care control arm. The primary endpoint is pCR (ypT0/is, ypN0), defined as the absence of invasive cancer in the breast and regional nodes at the time of surgery.[50] As I-SPY2 is modified intent-to-treat, patients receiving any dose of study therapy are considered evaluable; those who switch to non-protocol therapy, progress, forgo surgery, or withdraw are deemed 'non-pCR'. Secondary endpoints include residual cancer burden (RCB) and event-free and distant relapse-free survival (EFS and DRFS).[50]

Trial design

Assessments at screening establish eligibility and classify participants into subtypes defined by hormone receptor (HR) status, HER2, and 70-gene signature (Mammaprint) status.[51] Adaptive randomization in I-SPY2 preferentially assigns patients to trial arms according to continuously updated Bayesian probabilities of pCR rates within each biomarker signature; 20% of patients are randomly assigned to the control arm.[52] While accrual is ongoing, a statistical engine assesses the accumulating pathologic and MRI responses at weeks 3 and 12 and continuously re-estimates the probabilities of an experimental arm being superior to the control in each defined biomarker signature. An arm can be dropped for futility if the predicted probability of success in a future 300-patient, 1:1 randomized, phase 3 trial drops below 10%, or graduate for efficacy if the probability of success reaches 85% or greater in any biomarker signature. The clinical control arm for the efficacy analysis uses patients randomized throughout the entire trial. Experimental arms have variable sample sizes: highly effective therapies graduate with fewer patients in the experimental arm; arms that are equal to, or marginally better than, the control arm accrue slower and are stopped if they have not graduated, or terminated for lack of efficacy, before reaching a sample size of 75. During the design of each new experimental arm the investigators together with the pharmaceutical sponsor decide in which of the 10 *a priori* defined biomarker signatures the drug will be tested. Upon entry to the trial, participants are dichotomized into hormone receptor (HR) negative versus positive, HER2 positive versus negative, and MammaPrint High1 [MP1] versus High2 [MP2] status. From these 8 biomarker combinations (2 × 2 × 2) I-SPY has created 10 biomarker signatures that represent the disease subsets of interest (e.g., all patients, all HR+, all HER2+, HR+/HER2-, etc, for complete list see ref. [52] in which a drug can be tested for efficacy. Efficacy is monitored in each of these biomarker signatures separately and an arm could graduate in any or all biomarker signature of interest. When graduation occurs, accrual to the arm stops, final efficacy results are updated when all pathology results are complete. The final estimated pCR results therefore may differ from the predicted pCR rate at the time of graduation. Additional details on the study design have been published elsewhere.[9,53,54]

Eligibility

Participants eligible for I-SPY2 are women >18 years of age with stage II or III breast cancer with a minimum tumor size of >2 · 5 cm by clinical exam, or >2 · 0 cm by imaging, and Eastern Cooperative Oncology Group performance status of 0 or 1. HR-positive/HER2-negative cancers assessed as low risk by the 70-gene MammaPrint test are ineligible as they receive little benefit from systemic chemotherapy. Only HER2-negative patients were eligible for randomization to the pembrolizumab arm. Additional exclusion criteria for this arm included prior PARP inhibitor or immune checkpoint inhibitor therapy, use of immunosuppressive medications, or history of autoimmune disease.

Treatment

Participants in the control arm received standard NACT: 80 mg/m^2 intravenous paclitaxel weekly for 12 weeks, followed by 4 cycles of 60 mg/m^2 doxorubicin plus 600 mg/m^2 intravenous cyclophosphamide every 2 to 3 weeks (AC). Participants in the pembrolizumab

arm received standard NACT plus 200 mg intravenous pembrolizumab every 3 weeks for 4 cycles (weeks 1, 4, 7, and 10) concurrently with paclitaxel, as previously described.[9] For the first paclitaxel infusion, 20 mg dexamethasone was given and if no infusion reaction occurred, dexamethasone was reduced to 10 mg for week two, if no infusion reaction was observed with the first two treatments, dexamethasone was discontinued. Dose reductions and toxicity management were specified in the protocol. Adverse events were collected according to the NCI Common Terminology Criteria for Adverse Events (CTCAE) version 4.0. After completion of AC, patients underwent lumpectomy or mastectomy and nodal sampling, with choice of surgery at the discretion of the treating surgeon. All patients were screened for potential adrenal insufficiency before surgery with a morning serum cortisol level.

Trial oversight

I-SPY2 is conducted in accordance with the guidelines for Good Clinical Practice and the Declaration of Helsinki, with approval for the study protocol and associated amendments obtained from independent ethics committees at each site. Written, informed consent was obtained from each participant prior to screening and again prior to treatment. The I-SPY2 Data Safety Monitoring Board meets monthly to review patient safety.

METHOD DETAILS

Pretreatment biopsy processing

Core needle biopsies of 16-gauge were taken from the primary breast tumor before treatment. Collected tissue samples are immediately frozen in Tissue-Tek O.C.T. embedding media and then stored in -80°C until further processing. At the central I-SPY Laboratory an 8μM section is stained with hematoxylin and eosin (H&E) and pathologic evaluation performed to confirm the tissue contains at least 30% tumor. A tissue sample meeting the 30% tumor requirement is further cryosectioned at 30 μM, and samples are sent to Agendia on uncharged slides for gene expression profiling. As well, fresh-frozen pretreatment sections on uncharged slides are sent to the Petricoin Lab at George Mason University for protein/phospho-protein analysis. Finally, archival pretreatment formalin fixed and paraffin embedded (FFPE) tumor samples were sectioned at 4 μM and placed on charged slides for multiplex immunofluorence analysis.

Multiplex immunofluorescence tumor tissue profiling

Multiplex immunofluorescence (mIF) staining was performed on 4 μm sections from pre-treatment FFPE tumor blocks using the Opal 7-color fIHC kit (PerkinElmer, Waltham, MA). Optimization/validation of the mIF panels has been presented elsewhere.[55] None of the concurrent controls had FFPE tissue samples available (only frozen tissue). Since FFPE tissues were required for the mIF assays (assay protocols were optimized on FFPE, not frozen, tissue) FFPE samples from 48 non-concurrent controls from the latter part of the trial were utilized for these assays. These samples were matched to the concurrent controls based on receptor status, MammaPrint scores, and response to therapy. Staining was performed on a Ventana Discovery Ultra autostainer (Ventana/Roche, Indianapolis, IN). Each section was put through sequential rounds of heat-inactivation epitope retrieval (HIER), followed by incubation with primary antibody, then secondary horseradish peroxidase (HRP)-conjugated antibody. Primary antibody was visualized using different tyramide-linked Opal fluorophores for each primary antibody. Subsequent HIER steps removed bound antibodies before applying the next round of primary antibody, secondary HRP conjugate, and tyramide-Opal dye. After the final antibody sequence, sections were counterstained with DAPI and mounted with Vectashield fluorescence mounting medium (Vector Labs, Burlingame, CA). Primary antibodies and their paired Opal fluorphores are listed in Table S1. The sequence of antibody labeling was determined based on the target antigen's stability to repeated HIER steps. Each primary antibody was matched with an appropriate Opal TSA dye based on staining intensity and on the antigen's cellular localization to minimize potential signal crossover of co-localized targets.

mIF image analysis

Slides were imaged using the Vectra 3.0 slide scanner and visualized in Phenochart whole slide viewer (PerkinElmer). Fifteen to twenty multispectral images per each tumor biopsy were acquired using the 20X objective (200× absolute magnification). Spectral unmixing and cell segmentation was performed using the inForm quantitative pathology software (PerkinElmer). Cell phenotypes were assigned using openCyto, an R package providing an automated data analysis pipeline for flow cytometry. Cell fractions are reported as a percentage of total cell counts unless otherwise indicated. PD-L1 staining from mIF Panel 2 (antibody clone E1L3N) was assessed using the tumor proportion score (TPS) and the combined positive score (CPS). The TPS was defined as the number of PD-L1^{+} tumor cells divided by the total number of tumor cells multiplied by 100. The CPS was defined as the number of PD-L1^{+} tumor cells and PD-L1^{+} immune cells, divided by the total number of tumor cells multiplied by 100.

mIF spatial distribution analysis

To examine spatial relationships among specific cell phenotypes, we employed spatial point pattern analyses using a variety of R packages (phenoptr, spatstat, and divo). To study the spatial colocalization of cancer cells and immune cells, each mIF image was virtually divided into non-overlapping squares of 200 μm × 200 μm. This square size was chosen to be within the range of an estimated effective intercellular communication distance of ∼250 μm.[56] The number of cancer cells and immune cells within each

square was counted. To calculate colocalization of two cell types, A & B, we applied the Morisita-Horn (MH) similarity index[57,58] to the data using the R package 'divo'. The MH index ranges from 0 (no cells of type A share the same square with a cell of type B), indicating highly segregated cell populations, to 1 (an equal number of type A and type B cells in each square), indicating the two cell types are highly colocalized. MH indices were calculated for 21 pairs of cell types (Table S4).

To further examine spatial relationships, we utilized the nearest neighbor distribution function, *G(r),* to quantify the relative proximity of any two cell types. This function is a spatial distance distribution metric that represents the probability of finding at least one cell of a given type within a radius (*r*) of another cell type. The *G(r)* function computations were performed using the R package "spatstat". The area under the *G(r)* curve (AUC) for $r = 0$ to 20 μm was calculated and an overall Spatial Proximity Score, *SPS*, was obtained by averaging the AUC values obtained for each of the 15–20 ROI images for a given case. A large *SPS* indicates close proximity between the cell populations analyzed. The *SPS* is directional such that the score for T cells within 20 μm of tumor cells, denoted *Tm_T.SPS* will be different than the score for tumor cells within 20 μm of T cells (*T_Tm.SPS*). *SPS* scores were determined for 20 pairs of cell types (Table S4). The 20 μm interval was selected to build upon prior work in the field.[31,59,60] To put this into perspective, a lymphocyte has a diameter of ∼10 μm.

Gene expression profiling

Twenty to thirty 30 μM pretreatment tumor cryosections with 20–30% cellularity are collected and emulsified in 0.5mL Qiazol solution and sent to Agendia, Inc., for RNA extraction and gene expression profiling on Agilent 32K (Agendia32627_DPv1.14_SCFGplus with annotation GPL20078) or Agilent 44K (Agilent_human_DiscoverPrint_15746 with annotation GPL30493 (update of GPL16233)) expression arrays. As previously described,[22] for each array, the green channel mean signal was log2-tranformed and centered within array to its 75th quantile as per the manufacturer's data processing recommendations. All values indicated for non-conformity are NA'd out; and a fixed value of 9.5 was added to avoid negative values. Probeset level data per array were mean-collapsed to the gene level, and genes common to the two platforms identified. Expression data from the first ∼900 I-SPY2 patients distributed over the two platforms GPL30493 ($n = 333$) and GPL20078 ($n = 545$) were combined into a single gene-level dataset after batch-adjusting using ComBat (Johnson et al., 2007). Linear adjustment factors were derived from the larger ComBat operation, per platform, which can be used to batch correct raw files. The subsequent ∼90 samples (including patients from the pembrolizumab and a subset of controls), assayed on GPL20078, were batch corrected using these factors and added to the original set, yielding a normalized expression dataset comprising 987 patients x 19,134 (common) genes. These transcriptomic data and the associated batch correction model coefficients are available as part of the I-SPY2-990 mRNA/RPPA Data Resource in NCBI's *Gene Expression Omnibus* (GEO), SubSeries GSE194040 (mRNA) (https://www.ncbi.nlm.nih.gov/geo/query/acc.cgi?acc=GSE194040) and through the I-SPY2 Google Cloud repository (https://www.ispytrials.org/results/data).[22]

Protein/phospho-protein profiling

In addition, laser capture microdissection (LCM) was performed on pre-treatment biopsy specimens to isolate tumor epithelium for signaling protein and phospho-protein profiling by reverse phase protein arrays (RPPA) in the Petricoin Lab at George Mason University, as previously published.[61] Approximately 10,000 cells are captured per sample. RPPA samples were assayed on three arrays, each containing hundreds of samples from different arms of the trial quantifying up to 139 protein/phospho-protein endpoints (GPL28470) from hormone receptor ($n = 4$), HER family ($n = 14$), cell cycle/proliferation ($n = 20$), immune ($n = 18$), DNA repair deficiency (DDR; $n = 15$), AKT/mTOR/PI3K ($n = 7$), apoptosis/autophagy ($n = 10$), IGF1R ($n = 6$), TIE/ANG ($n = 4$), growth/survival/metabolism ($n = 22$) and RTK ($n = 19$) pathways, though this study only considers a (hypothesis-driven) subset of immune and DRD biomarkers. To remove batch effects we standardized each array prior to combining, by (1) sampling 5000 times, maintaining a receptor subtype balance equal to that of the first ∼1000 patients (HR + HER2-: 0.384, TN:0.368, HR + HER2+:0.158, HR-HER2+:0.09); (2) calculating the mean(mean) and mean(sd) for each RPPA endpoint; (3) z-scoring each endpoint using the calculated mean/sd from (2), as previously described.[61] Normalized and raw RPPA data over all endpoints for the 236 patients with RPPA analysis in this study are part of of the I-SPY2-990 mRNA/RPPA data resource deposited in NCBI's *Gene Expression Omnibus* (GEO) SubSeries GSE196093 (RPPA) (https://www.ncbi.nlm.nih.gov/geo/query/acc.cgi?acc=GSE196093) [22] and through the I-SPY2 Google Cloud repository (https://www.ispytrials.org/results/data).[22] The subset of immune- and DRD-related RPPA biomarkers analyzed in this study are also provided in Table S6 along with clinical data.

Biomarkers evaluated

126 total biomarkers from mIF, gene expression, and RPPA were assessed as specific predictors of response to Pembro. The mIF analyses yielded 61 immune-related biomarkers: 18 cell populations, 2 PD-L1 scores (CPS and TPS), and 41 spatial metrics (21 based on the Morisita-Horn index and 20 based on the nearest neighbor distribution function). There were 38 pre-specified expression signatures: 26 immune-related, 10 DNA damage response (DDR), 1 proliferation, and 1 hormone receptor (ER/PR). Finally, RPPA biomarkers included 17 immune-related and 10 DDR-related protein/phosphoprotein endpoints. Tables S2, S3, and S4 list these biomarkers, annotated by type, description, and references (as needed). Tables S5 (mIF) and S6 (mRNA and RPPA) contains per-patient scores for these 126 biomarkers and clinical variables (the latter is also available on GEO (https://www.ncbi.nlm.nih.gov/geo/query/acc.cgi?acc=GSE196096)) – for our study population.

CellPress
OPEN ACCESS

QUANTIFICATION AND STATISTICAL ANALYSIS

pCR association biomarker analysis

We assessed associations of biomarkers with response in the experimental and control arms using a logistic model (likelihood ratio (LR) test $p < 0.05$).[62] Analyses were also performed adjusting for HR status as a covariate, and numbers permitting, within receptor subsets. Markers are analyzed individually. Likelihood ratio p-values are adjusted for multiple hypothesis testing using the Benjamini-Hochberg method (Huang et al., 2009) with a significance threshold of BH $p < 0.05$, and considered descriptive rather than inferential. Analyses were performed in the computing environment R (v.3.6.3) using R Packages ‘stats’ (v.3.6.3), ‘lmtest’ (v.0.9–37), ‘rjags’ (v.4-10). Scripts are available upon request.

Cell Reports Medicine, Volume *5*

Supplemental information

Multi-platform biomarkers of response to an immune checkpoint inhibitor in the neoadjuvant I-SPY 2 trial for early-stage breast cancer

Michael J. Campbell, Denise M. Wolf, Christina Yau, Lamorna Brown-Swigart, Julie Wulfkuhle, Isela R. Gallagher, Zelos Zhu, Jennifer Bolen, Scott Vandenberg, Clifford Hoyt, Hidetoshi Mori, Alexander Borowsky, Laura Sit, Jane Perlmutter, Smita M. Asare, I-SPY2 Investigators, Rita Nanda, Minetta C. Liu, Douglas Yee, Angela M. DeMichele, Nola M. Hylton, Lajos Pusztai, Donald A. Berry, Gillian L. Hirst, Emanuel F. Petricoin, Laura van't Veer, and Laura Esserman

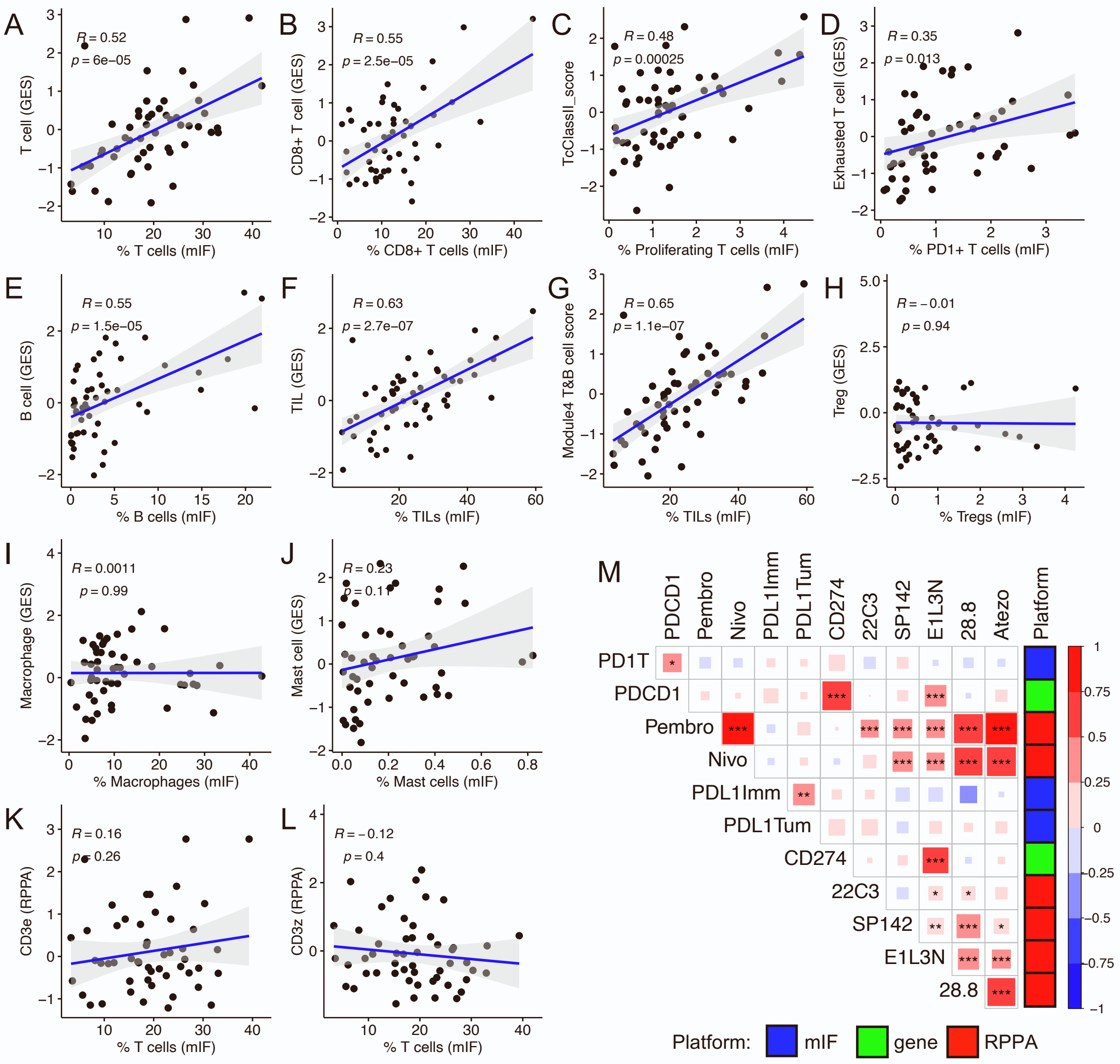

Figure S1. Correlations of immune cell densities measured on different platforms. Related to Figure 1. (A-J) Correlations of immune cells identified by mIF vs gene expression signatures (GES). (K-L) Correlations of immune cells identified by mIF vs RPPA. (M) Correlation matrix of PD-1/PD-L1 biomarkers. Pearson correlations; *p<0.01, **p<0.001, ***p<0.0001. Red square indicate positive correlation, blue squares indicate negative correlation. mIF markers --- PD1T: PD-1^{+} T cells; PDL1Imm: PD-L1^{+} immune cells; PDL1Tum: PD-L1^{+} tumor cells. Gene expression markers --- PDCD1: PD-1 gene; CD274: PD-L1 gene. RPPA markers --- Pembro (pembrolizumab) and Nivo (nivolumab): anti-PD-1 mAbs; 22C3, SP142, E1L3N, 28.8 and Atezo (atezolizumab): anti-PD-L1 mAbs.

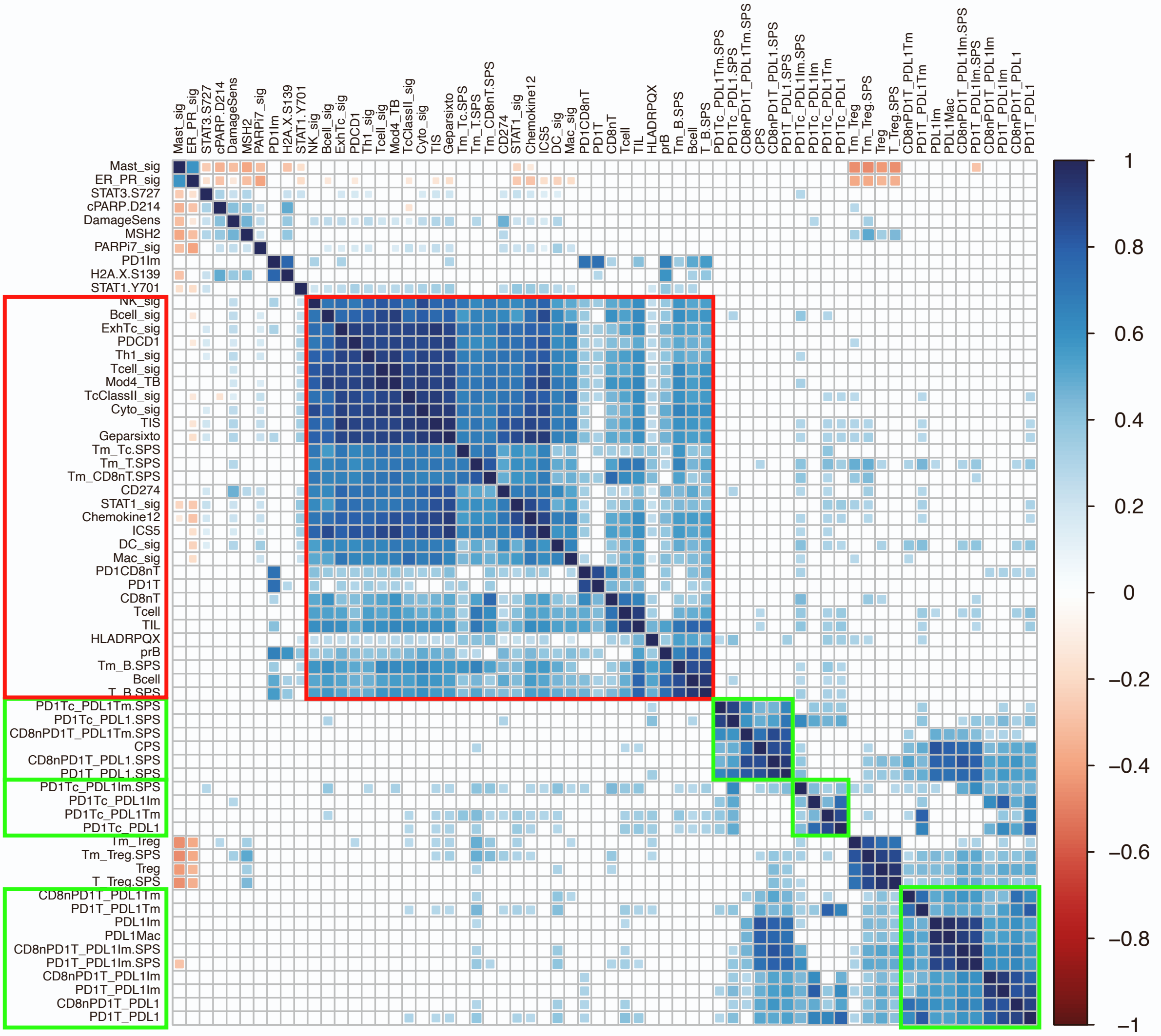

Figure S2. **Correlation matrix of biomarkers significantly associated with pCR in the pembro arm. Related to Figures 3 and 6 and Tables S2-S6.** Significant correlations (Pearson correlations, $p<0.05$) are shown in blue (positive correlation) or red (negative correlation). Red boxed area indicates cluster of immune cell gene expression signatures, immune cell densities by mIF, and immune cell population expression signatures. Green boxes indicate clusters of PD-1/PD-L1 related spatial metrics.

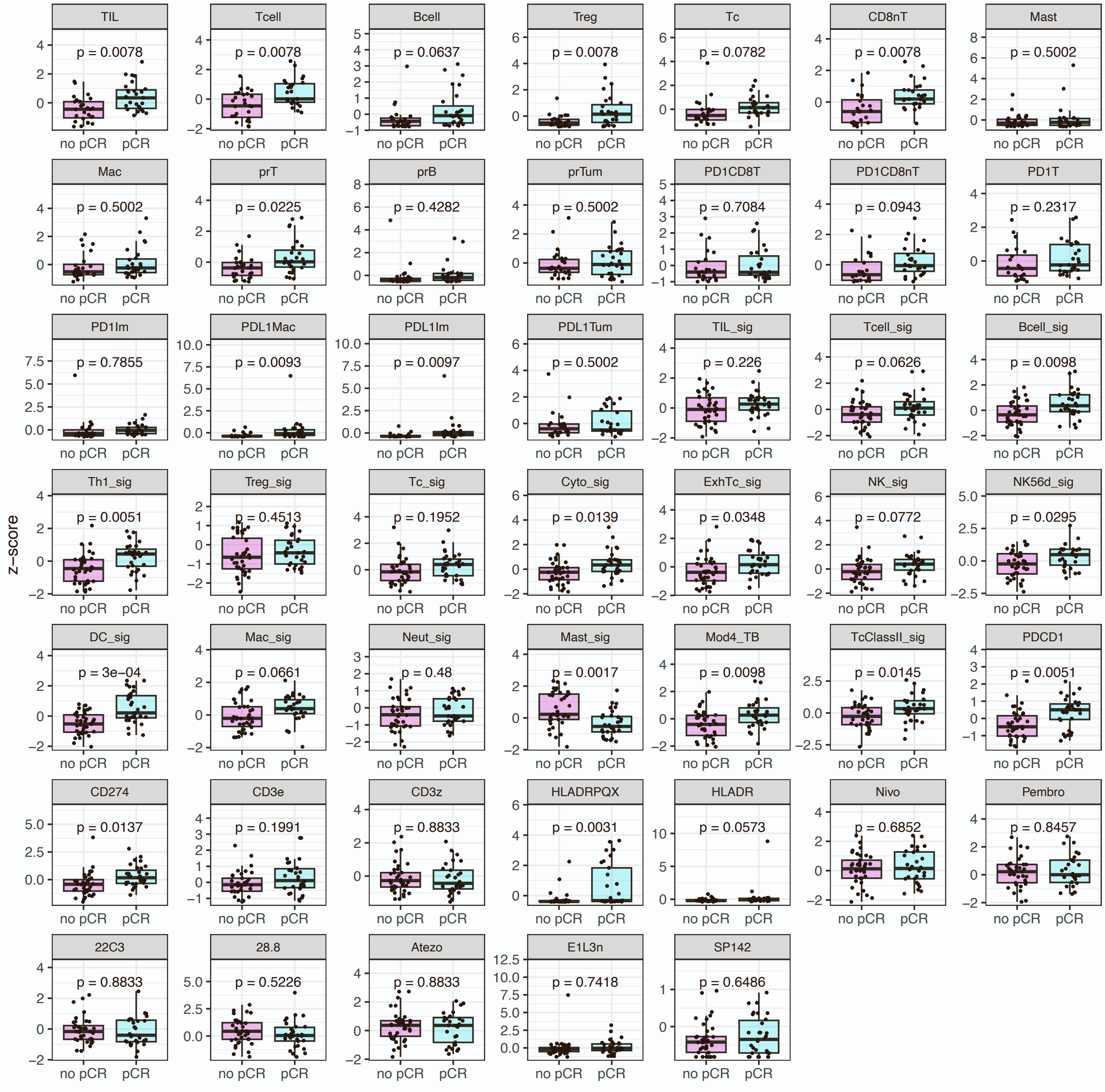

Figure S3. Associations of immune cell markers/populations identified by mIF, gene expression, or RPPA with response to ICB. Related to Figure 3 and Tables S2-S6. Boxplots illustrating the associations of immune cell densities (z-scored cell percentages), immune genes and gene signatures, and RPPA immune markers with response. Descriptions of mIF populations, gene signatures, and RPPA markers are given in Tables S2-S4. The data are depicted as individual dots for each sample, along with the median, first and third quartile. Statistical analysis was performed using the likelihood-ratio test. BH corrected p values shown.

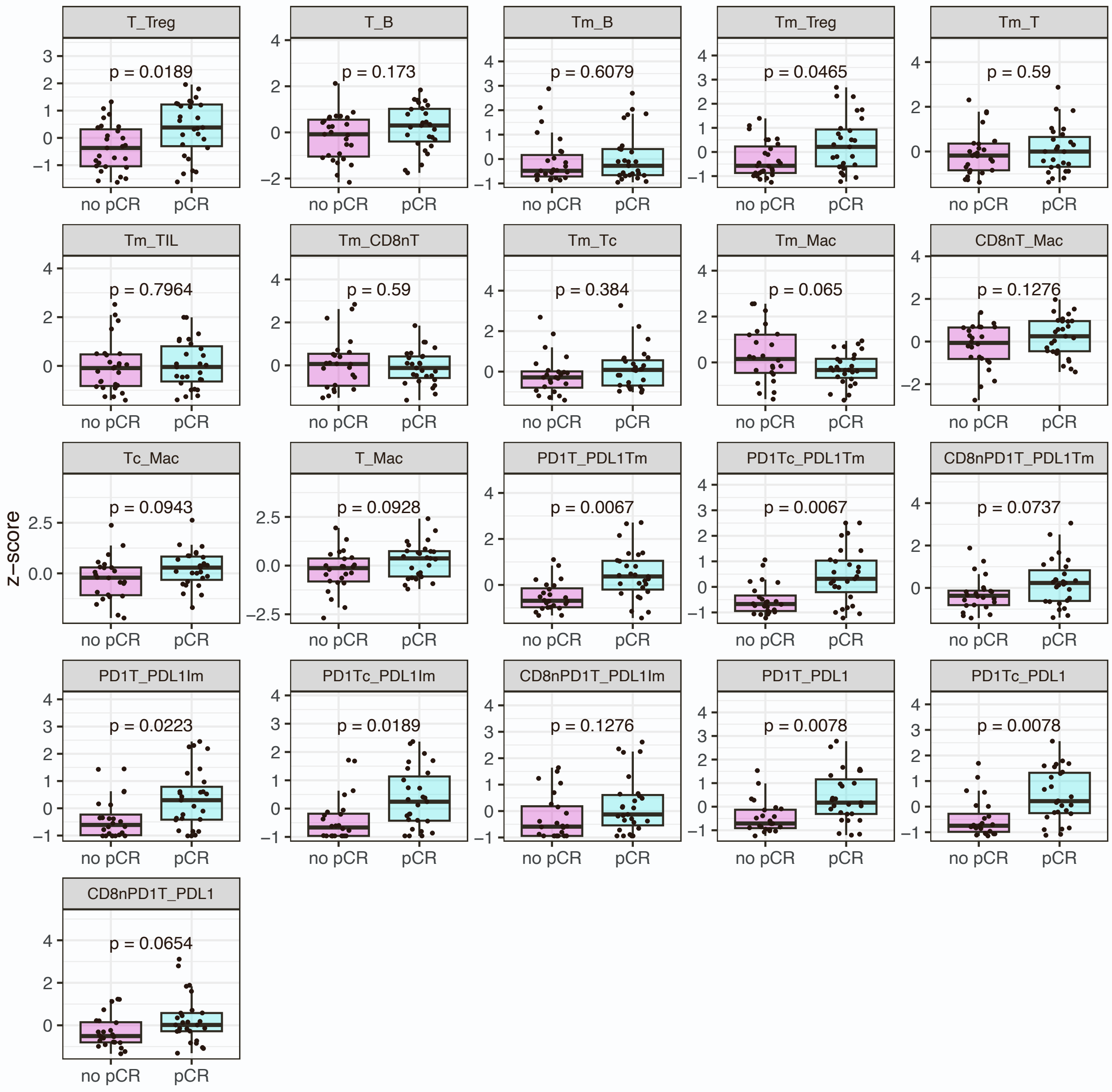

Figure S4. Associations of spatial colocalization of various cell types in the tumor microenvironment with response to ICB. Related to Figure 4 and Tables S4 and S5. Boxplots illustrating the associations of colocalization of cell pairs (Morisita-Horn indices; z-scored) with response. Descriptions of cell pairs are given in Table S4. The data are depicted as individual dots for each sample, along with the median, first and third quartile. Statistical analysis was performed using the likelihood-ratio test. BH corrected p values shown.

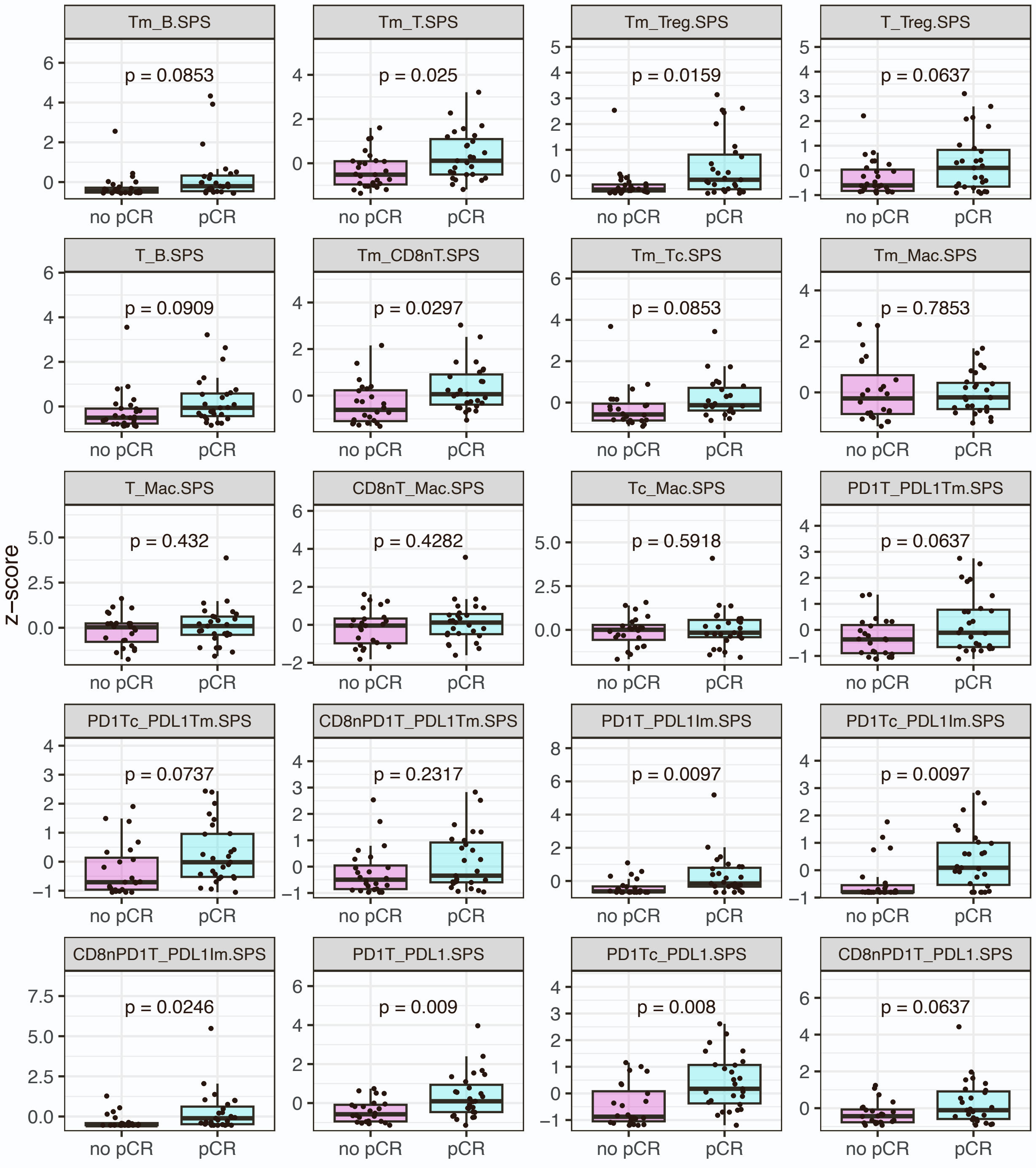

Figure S5. Associations of spatial proximity of various cell types in the tumor microenvironment with response to ICB. Related to Figure 5 and Tables S4 and S5. Boxplots illustrating the associations of spatial proximity scores (SPS) between cell pairs with response. Descriptions of cell pairs analyzed are given in Table S4. The data are depicted as individual dots for each sample, along with the median, first and third quartile. Statistical analysis was performed using the likelihood-ratio test. BH corrected p values shown.

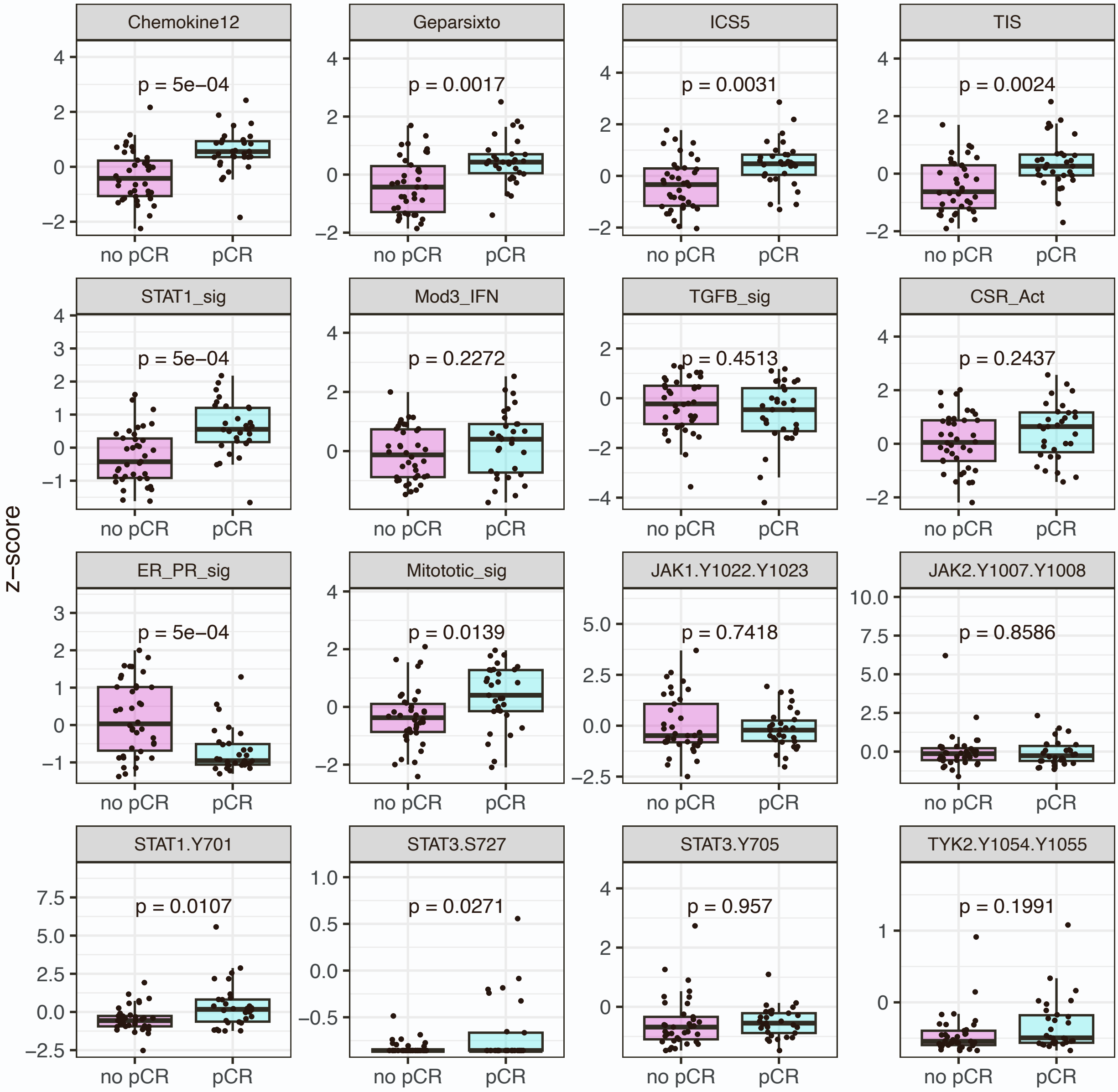

Figure S6. Associations of immune signaling pathways with response to ICB. Related to Figure 6 and Tables S2, S3, and S6. Boxplots illustrating the associations of signaling pathways measured by gene expression or RPPA with response. Descriptions of gene signatures are given in Table S2. Descriptions of RPPA markers are given in Table S3. The data are depicted as individual dots for each sample, along with the median, first and third quartile. Statistical analysis was performed using the likelihood-ratio test. BH corrected p values shown.

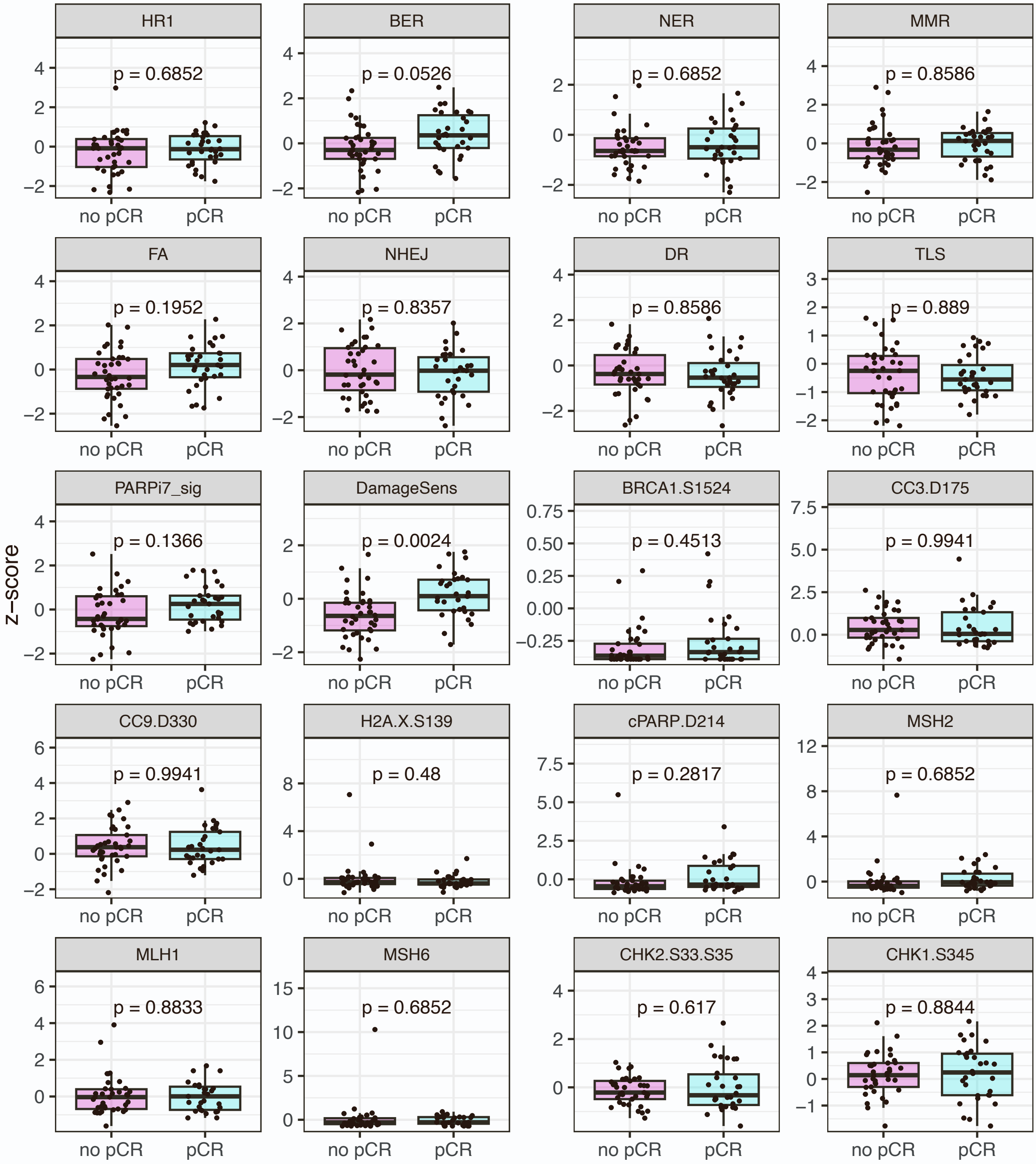

Figure S7. Associations of DNA damage and repair biomarkers with response to ICB. Related to Figure 6 and Tables S2, S3, and S6. Boxplots illustrating the associations of DNA damage and repair biomarkers measured by gene expression or RPPA with response. Descriptions of gene signatures are given in Table S2. Descriptions of RPPA markers are given in Table S3. The data are depicted as individual dots for each sample, along with the median, first and third quartile. Statistical analysis was performed using the likelihood-ratio test. BH corrected p values shown.

Supplemental Table S1. Details of primary antibodies and Opal fluorophores used in mIF. Related to Figure 2 and STAR Methods.

Panel 1

Marker	Clone	Antibody source	Opal TSA	RRID
FoxP3; regulatory T cell marker	SP97	Spring Biosciences	620	AB_10658507
pan-cytokeratin; epithelial cell marker	AE1/AE3	Dako	650	AB_2631307
CD20; B lymphocyte marker	L26	Ventana/Roche	540	AB_2335956
CD3; T lymphocyte marker	2GV6	Ventana/Roche	520	AB_2335978
Ki67; proliferation marker	30-9	Ventana/Roche	570	AB_2631262
CD117; c-Kit, mast cells	D3W6Y	Cell Signaling	690	AB_2799120

Panel 2

Marker	Clone	Antibody source	Opal TSA	RRID
PD-L1; immune checkpoint	E1L3N	Cell Signaling	620	AB_2687655
PD-1; immune checkpoint	EPR4877	Abcam	650	AB_2894867
CD8; cytotoxic T cell marker	4B11	Leica	690	AB_442068
pan-cytokeratin; epithelial cell marker	AE1/AE3	Dako	570	AB_2631307
CD68; macrophage marker	PG-M1	Dako	540	AB_2074844
CD3; T cell marker	2GV6	Ventana/Roche	520	AB_2335978

Supplemental Table S3. Reverse Phase Protein Array (RPPA) based biomarkers evaluated in this study. Related to Figures 3, 6, S2, S3, S6, S7, Table S6, and STAR Methods. DDR: DNA Damage Response.

Biomarker	Type	Description
CD3ε	cell population	CD3 epsilon chain
CD3ζ	cell population	CD3 zeta chain
HLADR	cell population	MHC class II moleculel; HLA-DR
HLADRPQX	cell population	MHC class II molecules; HLA-DR, HLA-DP, HLA-DQ, HLA-DX
28.8	cell population	PD-L1 (antibody clone 28.8)
Atezo	cell population	PD-L1 (atezolizumab)
SP142	cell population	PD-L1 (antibody clone SP142)
E1L3N	cell population	PD-L1 (antibody clone E1L3N)
22C3	cell population	PD-L1 (antibody clone 22C3)
Pembro	cell population	PD-1 (pembrolizumab)
Nivo	cell population	PD-1 (nivolumab)
STAT1.Y701	Immune signaling	Phospho-STAT1; signal transducer and activator of transcription 1
STAT3.S727	Immune signaling	Phospho-STAT3; signal transducer and activator of transcription 3
STAT3.Y705	Immune signaling	Phospho-STAT3; signal transducer and activator of transcription 3
JAK1.Y1022.Y1023	Immune signaling	Phospho-JAK1; Janus kinase 1
JAK2.Y1007.Y1008	Immune signaling	Phospho-JAK2; Janus kinase 2
TYK2.Y1054.Y1055	Immune signaling	Phospho-TYK2; JAK family non-receptor tyrosine protein-kinase
BRCA1.S1524	DDR	Phospho-BRCA1; breast cancer type 1 susceptibility protein
CC3.D175	DDR	Phospho-CC3; cleaved caspase 3
CC9.D330	DDR	Phospho-CC9; cleaved caspase 9
H2A.X.S139	DDR	Phospho-H2A.X; H2A histone family member X
cPARP.D214	DDR	Phospho-cleaved PARP; poly (ADP-ribose) polymerase
MSH2	DDR	MutS homolog 2, DNA mismatch repair protein
MSH6	DDR	MutS homolog 6, DNA mismatch repair protein
MLH1	DDR	MutL homolog 1, DNA mismatch repair protein
CHK1.S345	DDR	Phospho-CHK1, checkpoint kinase 1
CHK2.S33.S35	DDR	Phospho-CHK2, checkpoint kinase 2

Table S4. mIF based biomarkers evaluated in this study. Related to Figures 3, 4, 5, S2, S3, S4, S5, Table S5, and STAR Methods.

Biomarker	Type	Description
Tcell	cell population	T cells (CD3+)
Treg	cell population	Regulatory T cells (CD3+Foxp3+)
Bcell	cell population	B cells (CD20+)
Mast	cell population	Mast cells (CD117+ CK-)
TIL	cell population	Tumor infiltrating lymphocytes (Tell + Bcell)
Tc	cell population	Cytotoxic T cells (CD8+CD3+)
CD8nT	cell population	CD8-negative T cells (CD8-CD3+)
Mac	cell population	Macrophages (CD68+CK-)
prT	cell population	proliferating T cells (Ki67+CD3+)
prB	cell population	proliferating B cells (Ki67+CD20+)
prTum	cell population	proliferating tumor cells (Ki67+CK+)
PD1CD8T	cell population	PD-1 positive cytotoxic T cells (PD-1+ CD8+ CD3+)
PD1CD8nT	cell population	PD-1 positive CD8 negative T cells (PD-1+ CD8- CD3+)
PD1T	cell population	PD-1 positiveT cells (PD-1+ CD3+)
PD1Im	cell population	PD-1 positive immune cells (PD-1+ CK-)
PDL1Mac	cell population	PD-L1 positive macrophages (PD-L1+ CD68+ CK-)
PDL1Im	cell population	PD-L1 positive immune cells (PD-L1+ CK-)
PDL1Tum	cell population	PD-L1 positive tumor cells (PD-L1+ CK+)

CPS	immune score	PDL1 combined positive score: [(PDL1Im + PDL1Tum) / (tumor cell count)] * 100
TPS	immune score	PDL1 tumor proportion score: [(PDL1Tum) / (tumor cell count)] * 100

T_Treg	Morisita-Horn index	colocalization of non-Treg T cells & Treg
T_B	Morisita-Horn index	colocalization of T cells & B cells
Tm_B	Morisita-Horn index	colocalization of tumor cells & B cells
Tm_Treg	Morisita-Horn index	colocalization of tumor cells & Treg
Tm_T	Morisita-Horn index	colocalization of tumor cells & T cells
Tm_TIL	Morisita-Horn index	colocalization of tumor cells & TIL
Tm_CD8nT	Morisita-Horn index	colocalization of tumor cels & CD8- T cells
Tm_Tc	Morisita-Horn index	colocalization of tumor cells & CD8+ cytotoxic T cells
Tm_Mac	Morisita-Horn index	colocalization of tumor cells & macrophages
CD8nT_Mac	Morisita-Horn index	colocalization of CD8- T cells & macrophages
Tc_Mac	Morisita-Horn index	colocalization of CD8+ cytotoxic T cells & macrophages
T_Mac	Morisita-Horn index	colocalization of T cells & macrophages

Biomarker	Type	Description
PD1T_PDL1Tm	Morisita-Horn index	colocalization of PD-1+ T cells & PD-L1+ tumor cells
PD1Tc_PDL1Tm	Morisita-Horn index	colocalization of PD-1+ cytotoxic T cells & PD-L1+ tumor cells
CD8nPD1T_PDL1Tm	Morisita-Horn index	colocalization of CD8- PD-1+ T cells & PD-L1+ tumor cells
PD1T_PDL1Im	Morisita-Horn index	colocalization of PD-1+ T cells & PD-L1+ immune cells
PD1Tc_PDL1Im	Morisita-Horn index	colocalization of PD-1+ cytotoxic T cells & PD-L1+ immune cells
CD8nPD1T_PDL1Im	Morisita-Horn index	colocalization of CD8- PD-1+ T cells & PD-L1+ immune cells
PD1T_PDL1	Morisita-Horn index	colocalization of PD-1+ T cells & any PD-L1+ cell
PD1Tc_PDL1	Morisita-Horn index	colocalization of PD-1+ cytotoxic T cells & any PD-L1+ cell
CD8nPD1T_PDL1	Morisita-Horn index	colocalization of CD8- PD-1+ T cells & any PD-L1+ cell

Tm_B.SPS	Spatial Proximity Score	SPS score for tumor cells with a B cell within 20 um
Tm_T.SPS	Spatial Proximity Score	SPS score for tumor cells with a T cell within 20 um
Tm_Treg.SPS	Spatial Proximity Score	SPS score for tumor cells with a Treg cell within 20 um
T_Treg.SPS	Spatial Proximity Score	SPS score for T cells with a Treg cell within 20 um
T_B.SPS	Spatial Proximity Score	SPS score for T cells with a B cell within 20 um
Tm_CD8nT.SPS	Spatial Proximity Score	SPS score for tumor cells with a CD8- T cell within 20 um
Tm_Tc.SPS	Spatial Proximity Score	SPS score for tumor cells with a cytotoxic T cell within 20 um
Tm_Mac.SPS	Spatial Proximity Score	SPS score for tumor cells with a macrophage within 20 um
T_Mac.SPS	Spatial Proximity Score	SPS score for T cells with a macrophage within 20 um
CD8nT_Mac.SPS	Spatial Proximity Score	SPS score for CD8- T cells with a macrophage within 20 um
Tc_Mac.SPS	Spatial Proximity Score	SPS score for cytotoxic T cells with a macrophage within 20 um
PD1T_PDL1Tm.SPS	Spatial Proximity Score	SPS score for PD-1+ T cells with a PD-L1+ tumor cell within 20 um
PD1Tc_PDL1Tm.SPS	Spatial Proximity Score	SPS score for PD-1+ cytotoxic T cells with a PD-L1+ tumor cell within 20 um
CD8nPD1T_PDL1Tm.SPS	Spatial Proximity Score	SPS score for CD8- PD-1+ T cells with a PD-L1+ tumor cell within 20 um
PD1T_PDL1Im.SPS	Spatial Proximity Score	SPS score for PD-1+ T cells with a PD-L1+ immune cell within 20 um
PD1Tc_PDL1Im.SPS	Spatial Proximity Score	SPS score for PD-1+ cytotoxic T cells with a PD-L1+ immune cell within 20 um
CD8nPD1T_PDL1Im.SPS	Spatial Proximity Score	SPS score for CD8- PD-1+ T cells with a PD-L1+ immune cell within 20 um
PD1T_PDL1.SPS	Spatial Proximity Score	SPS score for PD-1+ T cells with any PD-L1+ cell within 20 um
PD1Tc_PDL1.SPS	Spatial Proximity Score	SPS score for PD-1+ cytotoxic T cells with any PD-L1+ cell within 20 um
CD8nPD1T_PDL1.SPS	Spatial Proximity Score	SPS score for CD8- PD-1+ T cells with any PD-L1+ cell within 20 um
